# Exosome-Based Therapeutics in Dermatology

**DOI:** 10.34133/bmr.0148

**Published:** 2025-05-09

**Authors:** Lanjie Lei, Shaoyu Zhou, Lingyao Zeng, Qiancheng Gu, Huaqian Xue, Fangyan Wang, Jiayin Feng, Shumao Cui, Liyun Shi

**Affiliations:** ^1^ Key Laboratory of Artificial Organs and Computational Medicine in Zhejiang Province, Institute of Translational Medicine, Zhejiang Shuren University, Hangzhou 310015, China.; ^2^ The Third Affiliated Hospital of Wenzhou Medical University, Wenzhou 325200, China.; ^3^ School of Food Science and Technology, Jiangnan University, Wuxi, Jiangsu 214122, China.

## Abstract

Exosomes (Exos) are tiny extracellular vesicles containing a variety of active biomolecules that play important parts in intercellular communication and influence the functions of target cells. The potential of Exos in the treatment of dermatological diseases has recently been well appreciated. This review highlights the constituents, function, and delivery of Exos, with a particular focus on their applications in skin therapy. Firstly, we offer a concise overview of the biochemical properties of Exos, including their sources, structures, and internal constituents. Subsequently, the biomedical functions of Exos and the latest advances in the extraction and purification of Exos are summarized. We further discuss the modes of delivery of Exos and underscore the potential of biomaterials in this regard. Finally, we summarize the application of Exo-aided therapy in dermatology. Overall, the objective of this review is to provide a comprehensive perspective on the applications and recent advancements of Exo-based approaches in treating skin diseases, with the intention of guiding future research efforts.

## Introduction

Exosomes (Exos) are small extracellular granular vesicles. These species are biologically functional and undergo secretion from the intracellular space to the extracellular environment, ultimately influencing the biological activities of recipient cells [1]. Initially, it was assumed that Exos functioned as a vehicle for the elimination of undesired intracellular proteins. Subsequently, Exos have been recognized for playing an important role in mediating inflammatory responses, facilitating cell proliferation, regulating the extracellular microenvironment, and eliciting the body's immune response [2,3]. Consequently, the potential of Exos as a viable substitute for cell therapy in disease treatment has become increasingly apparent.

However, the extraction and purification of Exos face major challenges due to their heterogeneity in terms of origin, size variation, and content diversity [4]. The presence of other extracellular vesicles (EVs), including microvesicles and apoptotic vesicles, can also complicate these processes due to their similar densities and structures, which can lead to sample contamination and reduced purity levels. In addition, the characteristics of lipoproteins overlap with those of Exos in terms of density and size, presenting similar purification and isolation challenges [5,6]. Currently employed methods for Exos purification include ultracentrifugation, ultrafiltration (UF), microfluidics, polymer precipitation, immunocapture, and exclusion chromatography, each tailored to a specific scenario [7,8]. Here, we provide an overview of Exos separation techniques, along with a comparative analysis of diverse sample types. This should aid readers in selecting an appropriate separation method that provides the optimal yield and purity for their system.

The characterization of Exos is necessary to assess the efficacy of Exos isolation and the quality of the resulting Exos. For example, protein content is frequently used as a reliable indicator for quantifying the purity of Exos [9]. However, performing such quantitative and phenotypic studies is challenging due to the small sizes and low protein content of Exos. In addition, many existing assays for Exos, such as nanoparticle tracking analysis (NTA) [10] and total protein assays [11], lack specificity and can be influenced by other proteins and cytokines, thereby compromising the accuracy and sensitivity of the results. Although antibody-based methods allow for the specific analysis of Exos [12], they are relatively insensitive to detecting rare Exos isoforms or low concentrations of Exos. Additionally, purification and centrifugation steps introduce additional experimental variables [13]. This review provides a summary of analytical identification techniques that map the different properties of Exos, along with a summary of some emerging techniques.

Recently, Exos have been explored in the development of personalized medicines owing to their homotypic targeting and self-recognition capabilities. However, in clinical applications, Exos present a new set of challenges, including low stability and poor targeting abilities. To improve the therapeutic efficacies of Exos, their surfaces can be engineered using homing peptides or ligands through a variety of engineering methods to impart them with targeting capabilities [14]. For example, receptor-specific molecules can be employed to bind Exos surface receptors, enabling their targeted delivery to the desired cells or tissues [15]. Furthermore, nanotechnology can be used to encapsulate drugs in nanoparticles and attach them to Exos, thereby enhancing their stability and targeting capabilities [16]. In addition to improving Exos delivery efficiency, for therapeutic purposes, it is also essential to ensure their safe and effective transport to the target site [3]. Therefore, the choice of biomaterial must be carefully considered when designing the delivery system. For instance, biomaterials such as chondroitin sulfate, alginate (Alg), and filipin protein have been widely used in carrier systems owing to their excellent biocompatibility and mechanical stability, which enable the effective protection and delivery of Exos [17]. Moreover, it is essential to achieve sustained release while maintaining adequate activity during treatment. Different formulations can therefore be selected based on specific requirements, such as loading capacity, to ensure that enough active Exos particles or liquid formulations are released at the disease site within an appropriate time frame. Therefore, additional efforts are necessary to overcome the current challenges in the clinical application of Exo, including optimizing the surface modification strategies to improve therapeutic efficacy, selecting appropriate biomaterials as loading systems to ensure safe and effective delivery, and exploring diverse formulations to meet the demands for sustained release.

Recently, increasing evidence has suggested that Exos can be extensively employed in the treatment of various dermatological conditions [18,19]. Exos can activate a variety of signaling pathways. By regulating the expression of relevant genes, Exos promote the activation and proliferation of target organ-related cells, which, in turn, repair damage [20]. Notably, antioxidants in Exos help reduce oxidative stress in the skin and improve skin texture. Additionally, Exos can penetrate the stratum corneum to reach the dermis and promote the synthesis of collagen and elastin, thereby slowing down the process of skin aging [21]. Exos not only are involved in skin physiological processes but also play a role in specific messaging when lesions occur in the skin microenvironment. They regulate the secretion of pro-inflammatory cytokines in the skin microenvironment and promote vascularization and collagen deposition in skin defects, thus maintaining the healthy state of the skin [22]. Moreover, exogenous Exos, especially those derived from mesenchymal stem cells (MSCs) [23,24], have immense potential for use in dermatological therapy and skin regeneration owing to their unique abilities to deliver biomolecules and modulate the cellular responses that contribute to restoring cellular function and immune homeostasis [25,26]. It is therefore evident that Exos hold great potential as diagnostic and therapeutic targets for the management of dermatologic disorders. Regarding the application of Exos to the skin, several reviews have summarized related topics [27,28], but the relationship between the multifunctionality of Exos and their application in treating dermatologic diseases has been less discussed. Additionally, the therapeutic effects of multifunctional Exo-aided therapy in dermatology have not been systematically analyzed and compared.

Here, we present an introduction to Exos, including their various sources, structures, internal components, and biomedical functions. We summarize the latest methods for extracting and purifying Exos, comparing their advantages and disadvantages. In addition, we present methods for identifying and modifying Exos for specific applications. Furthermore, we highlight biomaterials that can be used to deliver Exos and evaluate some common forms of Exos delivery that are currently being used in disease treatment. Finally, we discuss the use of Exos in dermatologic therapy, along with the challenges that these particles face in the clinical setting. Potential future research directions are also presented. In conclusion, this review aims to provide a comprehensive overview of Exos-based applications and recent advances in the treatment of dermatologic diseases, and inform research efforts in skin therapy and related systemic diseases.

## Exosomes

**Fig. 1.**
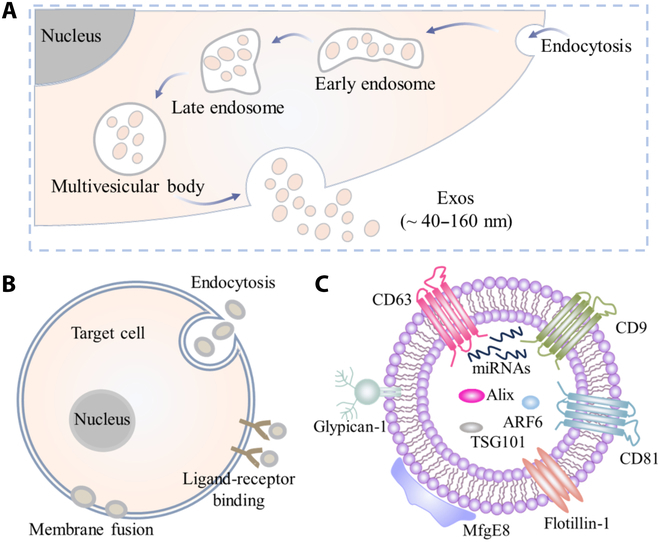
Introduction to exosomes (Exos). (A) Schematic of Exo production. (B) Forms of Exo action. (C) Exo biomarkers. The figure is reproduced with modifications from Ref. [31] (Copyright 2021, Gurung et al.).

Exos are formed during the formation of intracellular multivesicular bodies, and they are known to contain luminal vesicles encapsulated by a lipid bilayer membrane. These vesicles contain various biologically active molecules including small RNA, proteins, and metabolic molecules [29]. Exos measure approximately 40 to 160 nm in diameter [30] and are thought to originate from endocytosis [31,32] (Fig. 1A). They act as intercellular vectors [33], exerting their biological effects through membrane fusion, endocytosis, and binding to receptor ligands in target cells to achieve information transfer (Fig. 1B). In addition to their unique molecular compositions (Fig. 1C), several exosomal proteins have been identified as potential marker molecules for disease diagnosis [34,35]. For example, serum levels of miR-141 in prostate cancer patients differentiate between metastatic and localized disease. An analysis of Exos extracted from urine revealed a microRNA (miRNA) profile that can be used to detect urothelial carcinoma of the bladder [36]. Furthermore, Exos not only protect enzyme-sensitive substances from degradation but also possess intrinsic properties, such as small size, excellent biocompatibility, and notable CD47 surface expression, which enable them to evade phagocytosis and enhance their stability in vivo [37]. Consequently, Exos hold great promise as therapeutic agents owing to prolonged circulation time in the blood vessels. In addition, Exos can be used as a novel drug delivery system. The desired drug is loaded onto the surface or inside of Exos using exogenous or endogenous drug delivery technologies. As drug carriers, Exos can deliver drug molecules to target cells efficiently and specifically, providing a new concept and practical technology for the targeted therapy of diseases [38].

### Sources and heterogeneity of Exos

The heterogeneity of Exos can be attributed to variations in size, content, function, and cellular origin [39]. For example, size heterogeneity is primarily due to the nonuniform invagination of membranes during Exos biogenesis, while content heterogeneity is associated with different invagination and protein sorting mechanisms within various cell types [40]. In addition, source heterogeneity is related to the organ and tissue of Exos origin [41]. It has been shown that even within the same source, Exos can exhibit size and content variabilities due to differences in their subcellular structures and cellular states [42]. Cancer cell Exos, for example, demonstrate unique properties, including organ tropism and uptake by different cell populations [43].

Exos are widely found in the body fluids of various organisms and can also be released from cultured cells in vitro [44]. The role of Exos is determined by the specific cell type from which they originate. For instance, MSCs, macrophages, and tumor cells are all capable of releasing Exos. Due to this heterogeneity, variation exists in the abundance of markers across different Exos. Consequently, specific combinations of antibodies can be used to selectively isolate different types of Exos from samples. Furthermore, Exos derived from different sources exhibit variations in their yields, component contents, functions, and drug-carrying capacities, resulting in potential differences in their therapeutic efficacy. Therefore, the careful selection of appropriate sources for Exos isolation could minimize the side effects associated with drug delivery.

### MSC-derived Exos

MSCs exert their therapeutic effects by secreting neurotrophic factors and angiogenesis regulators, in addition to promoting nerve regeneration and neoangiogenesis [45]. They also possess immunomodulatory properties, suppress inflammation, and facilitate tissue regeneration in various disease models [46]. However, using MSCs as novel drug systems raises safety concerns due to potential unwanted differentiation after transplantation, the malignant transformation of MSCs, and alloimmune responses that can lead to graft rejection [47]. Research has shown that MSC Exos play an important role in facilitating intercellular communication, which serves as a pivotal mechanism in determining their therapeutic efficacy [48,49]. MSC Exos possess various advantages, such as convenient storage and transferability characteristics. Furthermore, MSC Exos are known to exhibit enkephalinase and insulin-degrading enzyme activities, which are responsible for reducing amyloid beta plaque deposition in Alzheimer's disease transgenic mice, thereby indicating their potential neurological effects [50]. Additionally, MSC Exos inhibit lymphocyte proliferation and induce anti-inflammatory lymphocyte differentiation. Moreover, MSC Exos may ameliorate inflammatory disorders by modulating DNA methylation levels to inhibit inflammation [51], thereby promoting the expression of molecules associated with epigenetic modification. In addition, MSC Exos inhibit inflammation and restore homeostasis in the body by activating relevant signaling pathways using their multiple RNA components. According to the miRNA profile of MSC Exos, cell proliferation is regulated by miR-191, miR-222, and miR-21, whereas angiogenesis is promoted by miR-222, miR-21, and Let-7a [52]. In addition, endothelial differentiation is promoted by miR-6087, muscle growth is facilitated by miR-494, and the inflammatory response is reduced by miR-181c [53].

Overall, MSC Exos can be regarded as a safe substitute for MSCs, serving as a novel cell-free therapy that modulates the inflammatory response and facilitates tissue repair and regeneration. Therefore, MSC Exos open up new therapeutic possibilities for treating various diseases and show great potential for clinical use.

### Macrophage-derived Exos

As a crucial element of the immune system, macrophages demonstrate high adaptability and are ​notable contributors to inflammatory reactions. They are mainly divided into M1 and M2 types. M1 macrophages can release numerous pro-inflammatory molecules that serve to enhance the inflammatory reaction, eliminate pathogens, and impede tumor development. Conversely, M2 macrophages release anti-inflammatory molecules that modulate the immune response while fostering tumor progression and spread. Macrophage Exos have been demonstrated to play crucial roles in the pathogenesis of numerous diseases. For example, M2 macrophages Exos can act as immunomodulators, enhancing the immune microenvironment of bone by activating the phosphatidylinositol-3-kinase (PI3K)/protein kinase B (AKT) pathway, facilitating the polarization of M1 macrophages to the M2 type, and accelerating diabetic fracture healing [54]. Moreover, M2 Exos have been shown to enhance the advancement and spread of tumors in colorectal, hepatocellular, and lung cancers [55]. In addition, since Exos possess characteristics that reflect their parent cells, differences can exist between Exos originating from different macrophage phenotypes. For example, Exos released from adipose tissue-derived M1 macrophages contain abundant miRNAs, such as miR-155 and miR-146a, which can induce insulin resistance in mice. Moreover, the secretion of miR-155-containing M1 Exos after a myocardial infarction exacerbates cardiac injury by acting on post-infarction vascular endothelial cells and inhibiting neoangiogenesis [56].

### Tumor cell-derived Exos

Tumor cells actively release a range of soluble biomolecules, including cytokines, chemokines, and growth factors, which play various roles in creating the environment around a tumor [57]. Tumor Exos contain various molecular components such as lipids, membrane-associated proteins, and RNAs. These components can modify the behavior of recipient cells and create pathways for malignant cell growth [58]. It is becoming increasingly clear that tumor Exos play important roles as immunomodulatory factors in the tumor microenvironment [59]. For example, Shang et al. [60] showed that novel colorectal cancer-derived Exos can act as pro-oncogenic molecules to promote proliferation, migration, invasion, and N1–N2 neutrophil differentiation of colorectal cancer cells through the miR-142-3p/miR-506-3p-TGF-β1 axis. In addition, renal cell carcinoma (RCC)-derived Exos promote tumorigenesis by altering activated lncRNA in sunitinib-resistant RCC (lncARSR)-induced macrophage polarization [61]. Tumor Exos could also be utilized as indicators for the early detection and diagnosis of disease, where targeting metastasis-associated Exos provides new ways for the development of effective antitumor therapies [62]. Following the modification of tumor cells, they may also facilitate cancer therapy [63]. In solid tumors, tumor Exos containing proteins and nucleic acids primarily enter vascular endothelial cells by endocytosis to stimulate neovascularization. Therefore, targeting Exos to induce angiogenesis holds great promise as a potential new approach for cancer therapy [64].

### Plant-derived Exos

Plant-derived Exos are small EVs secreted by plant cells, with similar contents to animal Exos but with a slightly larger particle size. Exos can be extracted from most plant cells, and as interest in plant Exos has increased in recent years, more types of Exos from a wider variety of sources are being studied [65]. Compared to animal Exos, plant Exos offer advantages such as low toxicity, low immunogenicity, high cellular uptake efficiency, high biocompatibility, and stability. They have the potential for large-scale production due to their low acquisition cost and technical requirements [66]. Studies have reported that plant Exos from various sources retain the biological functions of their original plants. Plant Exos can use their contents, such as small RNAs, miRNAs, long noncoding RNAs, etc., as extracellular messengers to mediate communication between plant and animal cells, participate in the defense against pathogens, and regulate the innate immunity of plants. They also act as carriers to transfer mRNAs, miRNAs, bioactive lipids, proteins, and other drugs. These features support biological activities such as anti-inflammatory, antitumor, and antiaging effects in animal cells. For example, Qi et al. developed a formulation by extracting Exo-like nanovesicles from aloe vera and mixing them with aloe vera hydrogel, which effectively regulated the balance between oxidative and antioxidative states. This improved the repair of natural barrier function in inflammatory lesions and wound sites [67], providing new insights into the rational design of Exo-like nanovesicle formulations based on natural plant-derived components.

## Structure and Composition of Exos

Exos are produced when cell membranes undergo inward budding, leading to the formation of endosomes within the cell. This involves a range of processes, and as a consequence of directional assembly and migration, fusion with the cell membrane occurs, resulting in the release of Exos. Exos are vesicular structures whose cholesterol- and diacylglycerol-rich membranes consists of a double layer of phospholipids and proteins, including the conventional transmembrane proteins, heat shock proteins, lysosomal proteins, tumor-sensitive gene 101, and fusion proteins [68]. The interior consists of a hydrophobic vesicle containing various bioactive molecules from the microenvironment and the cytoplasm of the parent cell [69]. Exos can carry signaling molecules, including growth factor receptors, kinases, and cytokines, which can interfere with the signaling and function of recipient cells. Consequently, the structures and compositions of these biomolecules are closely related to the resulting Exos function.

## Primary Functions of Exos

### Mediating cell-to-cell communication

As an important vehicle for intercellular communication, Exos play an indispensable role in maintaining the homeostasis of the organism [70]. These nanoscale vesicles carry an abundance of biomolecules that can accurately reflect the cellular state and transmit signaling molecules between cells, thereby regulating the gene expression and biological functions of target cells. In terms of biogenesis, Exos formation, content selection, and Exos release are finely regulated [71]. This precise molecular sorting mechanism ensures that Exo-mediated intercellular communication is highly specific. During signaling, Exos interact with target cells mainly through ligand-receptor binding, membrane fusion, or endocytosis. These interactions can trigger downstream signaling cascades that regulate key biological processes such as cell metabolism, proliferation, and differentiation [72]. Given the important role of Exos in intercellular communication, their role in disease pathogenesis has received increasing attention. Exos exhibit unique biological functions in tumor microenvironment regulation and immune response modulation [73]. These findings provide an important theoretical basis and application perspective for the development of novel Exos-based diagnostic markers and therapeutic strategies.

### Immunomodulatory function

Research has shown that Exos possess a range of immunomodulatory functions [74]. More specifically, Exos can regulate immune responses by carrying immunomodulatory molecules or messages, and such modulation can be either positive or negative [75]. For instance, certain Exos carry the inhibitory cytokines transforming growth factor-β (TGF-β) [76] and interleukin-10 (IL-10) [77], which suppress inflammatory and autoimmune responses and act as immunosuppressors. Among these cytokines, MSC Exos exhibit an immunomodulatory role by inhibiting lymphocyte proliferation while inducing their differentiation toward anti-inflammatory phenotypes [78]. In addition, adipose MSC Exos inhibit T cell differentiation, activation, and proliferation, while also inhibiting the production of the pro-inflammatory cytokine interferon-γ. Exos also influence the immune system and control immune cell functions. For instance, Exos can inhibit the activities of macrophages and dendritic cells to reduce the body's ability to fight pathogens [79], thereby exerting an immunosuppressive effect. Conversely, some Exos stimulate the activity of natural killer cells and CD8^+^ T cells to enhance their cytotoxicity against tumor cells and infectious agents, thereby acting as immune activators [80]. Exos therefore participate in ​notable complex regulatory mechanisms for the effective balancing and regulation of the immune response.

### Tissue repairing

**Fig. 2.**
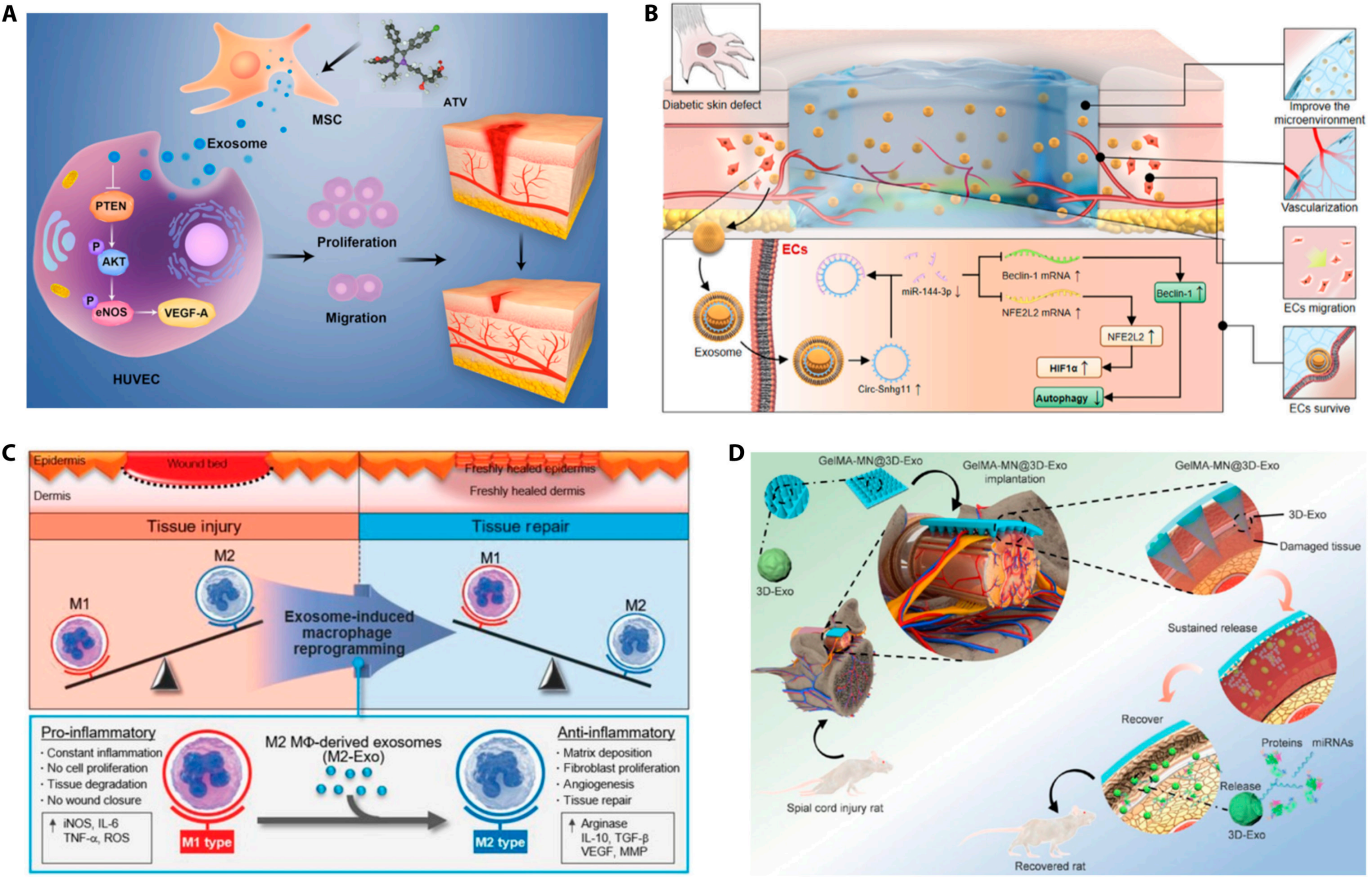
Exos for tissue repair. (A and B) Diagram outlining atorvastatin (ATV)-pretreated MSC Exos (A) [82] (Copyright ©2020, Yu et al.) and ADMSC Exos (B) [84] (Copyright ©2022, Acta Materialia Inc., published by Elsevier) promoted wound healing by increasing skin cell proliferation and migration. (C) M2 Exos accelerate wound healing by inducing M1-to-M2 conversion [90] (Copyright ©2019, *Advanced Science*, published by WILEY). (D) Diagram outlining the use of 3D Exo for the treatment of SCI in rats [97] (Copyright ©2022, *Nano Letters*, published by the American Chemical Society).

Exos include a variety of cell proliferation factors, pro-angiogenic factors, anti-inflammatory agents, and antioxidants, giving them ​crucial therapeutic potential in facilitating tissue repair [81]. Firstly, in the context of promoting cell proliferation and regeneration, MSC Exos have been shown to increase skin cell proliferation and migration and accelerate wound healing as shown in Fig. 2A [82]. Umbilical cord MSC (UCMSC) Exos have the potential to enhance fibroblast proliferation and collagen synthesis through the up-regulation of genes related to N-cadherin, cyclin-1, and collagen type 1, among others [83]. Additionally, adipose MSC (ADMSC) Exos improved wound healing and angiogenesis (Fig. 2B) in rats with spinal cord injuries (SCI) [84].

In terms of promoting revascularization, the vascular endothelial growth factor and fibroblast growth factor (FGF) in Exos can promote angiogenesis, improve tissue oxygenation and nutrient delivery, and accelerate wound healing [85]. Indeed, the promotion of vascular regeneration is a primary mechanism by which MSC Exos facilitate skin damage repair. This process primarily involves stimulating smooth muscle cell proliferation and migration, along with recruiting vascular endothelial cells to restore damaged blood vessels [86]. MSC Exos are also enriched with a diverse array of proteins and RNAs that are associated with angiogenesis, thereby stimulating the expression of numerous trophic factors [87].

In addition, inflammation is a crucial process in the body's defense system, recruiting neutrophils and macrophages to eliminate foreign particles and damaged tissues, ultimately aiding in skin regeneration [88]. Exos contain a variety of anti-inflammatory and antioxidant factors, which can inhibit the inflammatory response and degree of oxidative damage to wounds, thereby promoting wound healing [89]. M2 Exos have been shown to facilitate receptor macrophage conversion to the anti-inflammatory M2 phenotype (Fig. 2C) [90]. They also promote the activation, differentiation, and proliferation of B cells while inhibiting the proliferation of T cells. Moreover, they have the ability to transform activated T lymphocytes into regulatory T cells, leading to an immunosuppressive impact [48].

Exos also modulate cellular interactions and signaling through the transport of different biomolecules [91]. In addition, they have been demonstrated to regulate matrix synthesis, thereby promoting tissue repair and regeneration [92]. The extracellular matrix (ECM) is composed of various biomolecules, including collagen, elastin, polysaccharides, and proteoglycans, which are critical for ensuring its stability and biological function. By regulating both the matrix synthesis and degradation processes, Exos affect ECM composition and biological function [93,94]. In particular, certain biomolecules present in Exos, such as miRNAs and proteins, can translocate into target cells to modulate the genes and signaling pathways that are associated with matrix synthesis [95,96]. For example, the miR-21 and miR-29 components of Exos can increase collagen and elastin production by targeting genes that are involved in matrix synthesis. In addition, specific proteins such as TGF-β within Exos can promote cell growth and differentiation by influencing the synthesis of the ECM. Notably, it has been observed that traditional 2-dimensional (2D) culture methods result in a decline in the stemness of MSCs over time, thereby restricting the therapeutic potential of MSC Exos. Conversely, 3-dimensional (3D) culture conditions result in high therapeutic efficacies for Exos-based therapies, as presented in Fig. 2D [97]. Compared with 2D culture, 3D culture not only increased MSC cell stemness but also remarkably improved the efficiency of Exo production and more efficiently attenuated SCI-induced neuroinflammatory response and glial scarring.

### Neuromodulation

Exos use several mechanisms to regulate neuronal development, including tRNA transfer and the release of neurotrophic factors. More specifically, Exos release neurotrophic factors that regulate neuronal development by delivering them to target cells, and they also transfer RNA from specific genes into neurons via RNA-containing vectors, thereby regulating neuronal expression at the transcriptional and translational levels, ultimately affecting neuronal development [98]. Exos contain many neurotrophic factors, antioxidants, and other components that help neurons combat environmental stressors, such as oxidative damage and inflammation, thereby providing neuroprotection [99]. In this context, Ji et al. [100] found that MSC Exos attenuate excessive reactive oxygen species (ROS) production, lipid peroxidation, mitochondrial dysfunction, and apoptotic gene expression. In addition, Exos are known to contain numerous anti-inflammatory substances; therefore, it is possible that controlling the inflammatory response may promote Exos release, leading to potential implications for neuronal protection. By transporting molecules involved in the regulation of inflammation (e.g., regulatory T cells and interleukins), Exos can modulate the inflammatory responses of neurons, thereby influencing their function and survival. Notably, proteins encapsulated within the exosomal cargo may also be involved in modulating these responses. For example, exosomal neurotrophic factors maintain neuronal homeostasis by regulating apoptosis and inflammation. It has also been reported that Exos actively regulate immune cell responses toward neuronal inflammation. In particular, they can suppress monocyte polarization and activation, thereby attenuating the intensity of the inflammatory response. Moreover, Exos influence the interplay between neurons and glial cells to modulate the inflammatory response within the neural tissues. These vesicles induce immune responses that are essential for maintaining the integrity of the nervous system. In particular, under conditions where environmental factors such as ultraviolet (UV) light irradiation can induce damage to neurons, the immune-mediated release of Exos can provide effective protection [101].

## Extraction and Purification of Exos

### Differential centrifugation

Differential centrifugation (DC) is the most commonly used method for isolating Exos due to their small sizes [102]. DC is a separation method based on the difference in size and density of Exos compared to contaminant particles. Although DC is widely recognized as the most effective method for isolating Exos due to its ability to effectively remove cellular debris and contaminants, the application of DC across numerous cycles can potentially lead to a reduction in Exos retention within the sample, resulting in diminished yields. Moreover, high-speed centrifugation can induce fusion between pellets and contaminants or proteins, ultimately affecting the physical properties of Exos [103]. In addition, the DC method requires skilled operators, which hinders its application in the clinical environment [104].

To address these issues, the density gradient centrifugation method was developed to separate Exos from protein aggregates and nonmembranous particles based on differences in density and size. During this process, the vesicles and biomolecules are separated by adding separation media (e.g., sucrose and iodixanol) to generate a gradient system, such as that employed by D’Acunzo et al. [105]. They demonstrated that compared to a sucrose-based gradient, an iodixanol-based gradient exhibited superior efficacy in separating various categories of EVs, including distinct subpopulations such as microvesicles, Exos, and mitochondrial vesicles. Although the total EV yields were similar between the 2 gradients, the iodixanol-based gradient demonstrated enhanced separation capabilities.

### Ultrafiltration

UF is based on the retention of EVs by membranes with different pore sizes, and it is known to facilitate the separation of Exos by allowing the isolation of particles from 2 to 100 nm in diameter [106]. For example, when working with cell supernatants, Exos with diameters between 30 and 150 nm can be effectively filtered using a filter with a small pore size (0.22 μm), thereby excluding larger particles. The filtrate obtained after this step is collected and subjected to UF through a column (3 to 10 kD) to eliminate the smaller proteins present in the filtrate. In terms of its efficiency, economy, and simplicity, UF offers advantages over ultracentrifugation, in addition to allowing batch processing to be carried out. However, it should be noted that the narrow pores of UF membranes are prone to clogging, and the resulting excessive pressure can cause deformation of the EVs. As a result, the UF method tends to produce Exos in lower yields and purities. Thus, UF has been employed as a complementary technique to an ultra-isolation method and has also been combined with other purification methods to improve the purification results. For example, Gao et al. [107] compared UF-size-exclusion chromatography (UF-SEC) with the ExoQuick-TC precipitation method to isolate Exos from equal volumes of adipose tissue-conditioned medium. They observed that the UF-SEC approach gave higher yields along with acceptable purity levels. The use of UF in conjunction with other substances that exhibit specific affinities for Exos has also been considered to enhance the purification process. Xiang et al. [108] combined the UF technology with titanium dioxide micromaterials, initially using UF to decrease the urine volume and subsequently using the titanium dioxide micromaterials to enrich the urinary Exos through targeted interactions with the phosphate groups present on the surfaces of their phospholipid bilayers.

### Microfluidics

Currently, ultracentrifugation and UF methods are commonly employed to extract Exos from cells. However, these approaches are time-consuming and may compromise the structural integrity of the biological particles. In contrast, microfluidics provides a high-throughput approach to selectively trap Exos while allowing other components to pass through unaffected. This enables the efficient separation and analysis of Exos from low-density fluids [109]. Microfluidic devices with different isolation mechanisms, such as active (e.g., electric, magnetic, and acoustic fields) or passive (e.g., ciliated microcolumns, viscoelastic flow, and tangential flow filtration with polycarbonate membranes) mechanisms, play a crucial role in sample treatment [110]. Therefore, it is essential to design highly selective and affinity-based microfluidic devices to ensure the efficient extraction of Exos using this technique [111]. For example, Exos can be captured and enriched by acoustically activated nano-sieves in microfluidic chips. Ultrasonic excitation of the microspheres creates an interparticle Björk force that helps capture small nanoparticles (100 nm in diameter) as they pass through the filled bed. Consequently, the Exos can be isolated by binding to fluorescently labeled antibodies pre-immobilized on the surfaces of polystyrene microspheres, followed by detection using fluorescence microscopy. In addition, Zhao et al. [112] developed a 3D porous sponge microfluidic chip based on CD9 antibody functionalization to produce an efficient Exos enrichment platform. The authors found that this chip achieved an Exos capture efficiency of approximately 90%. However, despite the advantages of microfluidic methods, such as ease of integration, low sample consumption, and high-throughput capabilities, their widespread use is hampered by the complexity associated with device design, fabrication, and operation.

### Polymer precipitation

The polymer precipitation method involves using extremely hydrophilic polymers to create a hydrophobic microenvironment by binding to the water molecules surrounding the Exos. This results in the precipitation of Exos, which can then be isolated through low-speed centrifugation. Polyethylene glycol (PEG) is one of the most widely used polymers for this purpose. In this approach, the sample is first centrifuged at a low speed to remove cellular debris. PEG is then added to the supernatant, and the Exos are concentrated by centrifugation. Washing and additional centrifugation steps are subsequently performed to remove the PEG and any other contaminants. Notably, this approach is simpler to use compared to the widely used ultracentrifugation method, featuring shorter analysis times and not requiring specialized equipment. However, it can be difficult to completely remove the polymers introduced during precipitation, which can also contain numerous impurities, resulting in relatively low purities and recoveries. These factors can hinder subsequent experimental analyses [104]. To address these issues, Antopolsky et al. [113] used a chemical coprecipitation technique to prepare magnetic Fe_3_O_4_ nanoparticles coated with PEG. By controlling nanoparticle movement, they captured proteins from the serum and purified the Exos via magnetic precipitation.

### Immunoaffinity capturing

Immunoaffinity capture methods employ antibodies to selectively capture Exos that express specific markers. Compared to the commonly used ultracentrifugation approach, these techniques yield notably higher Exos purities [114]. However, if the antibody cannot be readily dissociated from the vesicle following precipitation, it may compromise the integrity of the Exos. Consequently, many solutions have been developed to meet this challenge. Cai et al. employed the host–guest interactions between β-cyclodextrin (β-CD) and 4-aminoazobenzene to conjugate antibodies with superparamagnetic nanoparticles to provide immunoaffinity superparamagnetic nanoparticles. This method enabled the efficient capture of Exos in cell culture supernatants and body fluids with a capture rate of 80% [115]. The subsequent competitive elution of the captured Exos was achieved using α-CD, resulting in a release rate of 86%. Furthermore, Lim et al. employed an agarose gel-based method using antibody cocktail-conjugated magnetic nanowires to efficiently isolate homogeneous populations of Exos from small plasma samples obtained from patients with cancer. Although they demonstrated rapid isolation with relatively high purities [116], this immunoaffinity capture approach only captured Exos that were successfully recognized by the antibodies, resulting in low yields.

### Size-exclusion chromatography

In SEC, smaller molecules are retained due to their permeation into the stationary phase, while molecules larger than the pore size of the stationary phase retain in the mobile phase and are eluted earlier than the smaller molecules. As previously reported, Exos extracted using SEC exhibit higher Exos recoveries compared to those obtained through ultracentrifugation [117]. This method offers numerous advantages, such as good cost-effectiveness, reproducibility, and high speed, thereby serving as an alternative for clinical samples in proteomics studies [118]. SEC is commonly employed for EV analysis in blood samples. For instance, Karimi et al. [119] described using SEC with a density pad to facilitate the separation of EVs from lipoproteins with lower densities. Furthermore, it has been found that loading floating EVs onto an SEC column allows for their separation from smaller soluble proteins and lipoproteins, thereby enabling detailed plasma EV proteome analysis. This combined approach expected to provide valuable insights into future Exos purification methods. However, it should be noted that SEC cannot differentiate between Exos and microbubbles of similar sizes; hence, it is often used in conjunction with other techniques [120].

## Characterization of Exos

The qualitative and quantitative characterization of Exos is commonly employed to evaluate the isolation effectiveness and Exos quality based on biomolecule yields and purities. For example, the evaluation of protein content is a common means to quantify Exos. Currently, fluorescence correlation spectroscopy (FCS), mass spectrometry, and marker protein assays are commonly used to identify specific protein markers. In addition, mass spectrometric analysis of the lipid compositions of Exos membranes can successfully distinguish between different subtypes of Exos. The ratio of the targeted lipids to the total Exos can be used as a metric to assess the purity of specific Exos. In contrast, quantitative analysis typically involves particle-counting methods, such as NTA and dynamic light scattering (DLS) [121].

### Particle size detection

NTA is a high-throughput visualization technique used to monitor the Brownian motion of particles in suspension, enabling the determination of the mean size, mode, and size distribution of Exos. The measurement process typically requires only a few minutes to complete, and in certain cases, the sample can be recovered after measurement (i.e., when sample dilution is not required for measurement) [122]. However, NTA has several limitations, including a lack of specificity toward Exos, inadequate discrimination between some species (e.g., nanoparticles, large protein aggregates, and biological vesicles), and potential interference from lipoproteins and protein aggregates during the quantification process.

Similar to NTA, DLS estimates the particle size by analyzing the scattered light resulting from the Brownian motion of particles. However, compared to NTA, DLS offers advantages in terms of its user-friendly nature and its capability to analyze small sample volumes [122]. Additionally, DLS is noninvasive, allows the rapid analysis of large numbers of particles, and permits complete sample recovery at a low cost. However, DLS also has some limitations. For example, the scattered light intensity is affected by the sixth power of the particle diameter, rendering detection challenging for smaller particles, and leading to biased data favoring larger sizes when mixtures are present in suspensions. Consequently, inaccurate particle counts may be obtained for samples with low purities [123]. Furthermore, nonexosomal contaminants measuring 30 to 100 nm (e.g., protein aggregates and lipoproteins) can be mistakenly identified as Exos using DLS methodology, resulting in an overestimation of the Exos quantity measured by this technique. DLS can therefore be considered suitable for monodisperse systems, but unsuitable for complex Exos samples with wide size ranges. Moreover, it cannot determine the Exos concentration accurately or differentiate contaminating proteins from similarly sized particles that are combined with the Exos.

### Fluorescence correlation spectroscopy

FCS is a high-throughput statistical technique used for characterizing molecular counts and diffusion coefficients [124]. FCS involves irradiating a small volume of a fluorescently labeled sample (e.g., DiR, DiO, DiD, FM 4-64, CFSE, PKH-26, or PKH-67) with a laser to observe the fluctuations in fluorescence intensity caused by Brownian motion. The change in fluorescence intensity over time is subsequently used to estimate the particle concentration. However, it should be noted that FCS has several limitations because the employed lipophilic fluorescent dyes are not specific to Exos, and as a result, they stain lipid-containing particles and vesicles present in the sample. Moreover, in salt-containing buffers, these lipid dyes tend to form micelles or aggregates that partially overlap with other components of the Exos, making their removal challenging [125]. To address this issue and enable the high-throughput screening and characterization of Exos based on antibody–vesicle interactions, FCS can be considered an innovative approach. Compared to other methods, such as flow cytometry or protein blotting, which operate on similar principles, FCS represents a sensitive high-throughput approach [125].

### Mass spectrometry

Mass spectrometry is a powerful qualitative and quantitative analytical method that is commonly used to identify and quantify a wide range of clinically relevant analytes. Following ion sorting based on their mass-to-charge ratio (*m/z*), the ions are measured and displayed on a mass spectrum. This technique enables the systematic characterization of Exos-specific proteins when combined with bioinformatics. Consequently, such approaches are commonly employed in the analysis of cancer-related biomarkers, such as Exos derived from infected cells [126]. These Exos can be sourced from serum [127] or urine [128], which holds notable importance in the context of early cancer detection and prognosis.

### Marker protein detection

Immunoassays are analytical techniques used to identify polyclonal or monoclonal antibodies specific to any antigens present in each given sample. Exos possess a variety of proteins on their membranes that can serve as potential markers (e.g., CD63, Tsg101, CD9, and CD81). However, although marker protein assays allow for the qualitative or quantitative analysis of these markers in Exos, they are complicated and time-consuming procedures with variable detection efficiencies.

Although flow cytometry is rapidly advancing, the majority of instruments have a detection limit between 200 and 500 nm, which falls short of the size range of EVs [129]. Another challenge arises when high concentrations of Exos are present or when aggregation occurs during isolation, as flow cytometry often struggles to accurately identify and distinguish multiple vesicles from a single entity, leading to potential inaccuracies in the results [130]. Additionally, the fluorescence signal obtained by flow cytometry can be influenced by cellular debris and cytoplasmic proteins. Therefore, precise quantification of the particle counts using flow cytometry necessitates samples with high purity. To address these commonly encountered issues, a viable solution involves the use of latex beads coated with monoclonal antibodies that specifically bind to proteins on the Exos surfaces. Once immobilized on the surface of the bead, the fluorescently conjugated antibodies target the antigens expressed on the Exos surfaces. This not only allows for high-throughput analysis but also permits easy quantification based on antigen expression.

Western blotting is also commonly employed for Exos analysis due to its simplicity and capability to identify both surface and internal proteins [131]. Moreover, the enzyme-linked immunosorbent assay approach, which is an immunolabeling technique based on antibody recognition of peptides and proteins, enables the detection of protein markers and the quantification of specific antigens present on the Exos [132]. The high specificity and rapidity associated with this assay allow for the precise analysis of marker proteins [133].

### Electron microscopy

Transmission electron microscopy (TEM) and scanning electron microscopy (SEM) are commonly employed techniques for assessing EV morphologies [134]. Both approaches utilize electron beams to generate high-resolution images of submicron particles; however, the distinction between these 2 methods lies in their respective electron detection protocols. In SEM, the electrons are scattered upon interaction with the sample particles. These scattered electrons are subsequently captured and detected, resulting in particle imaging. In TEM, noninteracting electrons pass through the sample and are detected by a fluorescent screen; dark areas or shadows on the phosphor screen generated by the sample particles produce the final image. Overall, electron microscopy enables direct observation of the Exos morphology and structure; SEM facilitates surface structure examination whereas TEM provides insights into the internal structure and information regarding the particle size distribution. However, due to its intricate operation and more demanding sample preparation requirements, TEM is not suitable for rapid, high-volume measurements. As an alternative approach, atomic force microscopy accounts for less than 5% of Exos studies compared with the widely employed TEM; its primary application lies in morphological evaluations [135].

## Targeted Modification of Exos

### Genetic engineering modification

Genetic engineering modification involves the fusion of gene sequences from target molecules with the gene sequence of a specific Exos membrane protein [136,137]. The surface protein LAMP-2B is frequently utilized in Exos research. For instance, Liang et al. [138] fused the chondrocyte affinity peptide to LAMP-2B by gene editing to form a hybrid Exos, endowing this species with the ability to target chondrocytes in the treatment of osteoarthritis. Additionally, transfection of the relevant plasmids into donor cells is known to allow the expression of Exos containing target proteins. In this context, Du et al. [139] successfully constructed a CD47-overexpressing Exos loaded with the inducer erastin and the photosensitizer rose bengal, which evaded phagocytosis by the mononuclear phagocytic system and exhibited increased distribution levels in tumors, thereby inducing ferroptosis in tumor cells.

### Chemical surface modification

Chemical modification involves the binding of natural or synthetic ligands to Exos proteins through coupling reactions or lipid assembly processes. Click chemistry is a widely used technique for the surface modification of Exos, with the acetylene group stacking reaction being one of the most common click chemistry reactions employed in Exos modification. For instance, Kang et al. used copper-free click chemistry to covalently attach myocardial-targeting peptides to the surfaces of human peripheral blood-derived Exos and attached cholesterol-modified siRNAs to the Exos surfaces. Consequently, the targeted delivery of siRNA was achieved, and cardiac function was remarkably improved [140].

### Physical handling

Various physical methods such as coincubation, electroporation, freeze–thaw cycling, saponin treatment, ultrasound exposure, and extrusion techniques have been shown to enhance the carrier functions of Exos. Although coincubation is a straightforward approach for loading hydrophobic or small-molecule drugs into Exos, it is not suitable for loading hydrophilic drugs. Electroporation facilitates the entry of drugs and nucleic acids into Exos by creating transport channels across the exosomal membrane, whereas ultrasound irradiation induces shear deformation of the exosomal membranes to enable efficient drug delivery. Furthermore, compression ruptures the Exos membrane to facilitate cargo loading, whereas freeze–thaw cycling modifies both the structure and functionality of the Exos membrane to ensure effective cargo encapsulation. Notably, all these physical methods (except for coincubation) introduce additional substances into the Exos system to varying degrees and affect its membrane architecture.

For example, Yang et al. [141] introduced plasmid DNA into different types of cells and then applied focused, temporary electrical stimulation to induce Exos with transcribed mRNAs and specific peptide release. In another study, Shi et al. [142] evaluated the incubation, sonication, extrusion, freeze–thaw cycling, saponin-assisted, and electroporation methods to enhance drug loading into Exos. They found that the Exos prepared by extrusion had the largest drug loading capacity, which was 2.45 times higher than that prepared by direct incubation. Minimal morphological changes were observed in the Exos obtained by the freeze–thaw cycling method.

Exo–liposome hybridization can also be used to optimize the properties of the Exos surface to increase their half-lives in the bloodstream, ultimately enhancing Exos uptake by the target cells in vivo [143]. In this context, Sato et al. [144] fabricated hybrid Exos by merging membranes with liposomes via the freeze–thaw technique and demonstrated that the transport capabilities of Exos could be altered through membrane fusion. Furthermore, Li et al. [145] fused CD47-expressing tumor Exos and liposomes to form hybrid nanoparticles for the codelivery of miR-497 and triptolide to achieve targeted treatment for ovarian cancer.

## Biomaterials for Exos Delivery

Although Exos are widely utilized in disease treatment research, their clinical application is hindered by low yield and efficiency, as well as a limited duration of delivery. Specifically, the rapid clearance of Exos by the immune system remarkably reduces their retention time in vivo, compromising their therapeutic efficacy [146]. To address these challenges, numerous studies have explored combining Exos with various biomaterials to enhance tissue therapeutic effects and repair efficiency. By maintaining Exos activity, prolonging their action, and promoting controlled release, these biomaterials serve as effective carriers. Exos are combined with biomaterials primarily through surface grafting, internal encapsulation, and the incorporation of nanoparticles to leverage the strengths of both components for more efficient delivery of therapeutics [147]. Biomaterials commonly used for Exos loading include chondroitin sulfate, hyaluronic acid (HA), and chitosan (CS).

### Chondroitin sulfate

Chondroitin sulfate is a glycosaminoglycan with excellent biocompatibility, known to possess anti-inflammatory, antioxidant, and antidegradation properties. It is abundantly present in various mammalian tissues, including bone, cartilage, skin, neural tissues, the ECM, and blood vessels [148]. As polysaccharides are abundant natural compounds containing diverse reactive groups, they can be easily modified to prepare chondroitin sulfate-based drug carriers for extensive biomedical applications. The use of chondroitin sulfate for Exos delivery has been considered an innovative approach, as demonstrated by Nikhil and Kumar [149], who developed cryogel cartilage scaffolds composed of chondroitin sulfate, gelatin, and chondroitin sulfate as biomaterials for efficient Exos delivery in the context of cartilage repair.

### Alginate

Alg is a linear polysaccharide widely used in tissue engineering due to its excellent biocompatibility, cost-effectiveness, and similar properties to the human ECM [150]. In addition, Alg hydrogels can be gelled with divalent cations under mild conditions, rendering them potential encapsulation materials for loading Exos [151]. The combination with hydrogels prevents premature Exos degradation and provides a sustained and pronounced therapeutic effect by ensuring a concentrated dose of Exos; this is achieved by placing the Exos-containing hydrogel directly at or near the target site. For example, Zhang et al. [152] prepared an injectable Alg-based composite gel by combining an Alg hydrogel with Exos secreted by dendritic cells. This composite gel effectively improved the therapeutic effects of Exos in enhancing cardiac function in a mouse myocardial infarction model. In another study, Gan et al. loaded MSC Exos inside sodium Alg microspheres, which created a moist environment to maintain the MSC Exo activity. Subsequently, the MSC Exos were encapsulated with gelatin to protect them from degradation in the acidic and enzymatic environment of the gastrointestinal tract, ensuring their release at the appropriate site to fulfill their biological function. Notably, this material has been used in therapeutic models of inflammatory bowel disease [153].

### Silk fibroin

Silk fibroin (SF) is a naturally occurring protein polymer that has been successfully employed as a drug delivery system [154]. SF exhibits exceptional mechanical strength, slow in vivo biodegradability, and high biocompatibility [155]. Similar to Alg salts, SF can be loaded with sensitive drugs (proteins and nucleic acids) through a gentle aqueous process [156]. For example, Rui et al. [157] employed in situ photocrosslinking of filipin protein hydrogels (Exos@SFMA) encapsulating MSC Exos to modulate the immune microenvironment in rheumatoid arthritis. In addition, Han et al. [158] demonstrated that delivering miR-675 via filipin protein hydrogel-encapsulated stem cell Exos prevented age-related vascular dysfunction in mouse hindlimbs. In another study, Li et al. [159] proposed engineering Exos that were modified to carry miR146a attached to a filipin protein patch for promoting diabetic wound healing. Therefore, SF has gained ​notable attention in the field of wound healing due to its potent antimicrobial properties.

### Hyaluronic acid

HA is the most abundant acidic mucopolysaccharide in the skin, synthesized by fibroblasts and keratinocytes present in skin tissue. HA plays a crucial role in the ECM, providing physical support while also participating in the regulation of cellular functions (e.g., promoting cell adhesion and angiogenesis) [160]. HA-based hydrogels and scaffolds have been used to improve the therapeutic efficacies and stabilities of Exos [161]. For example, Liu et al. [162] showed that a combination of HA with AMSC Exos could expedite wound healing by enhancing epithelial regeneration and vascularization. Furthermore, Derkus [163] integrated cardiac Exos with HA hydrogels to demonstrate their potential in promoting cardiac tissue regeneration. Moreover, due to its inherent ability to bind specifically to tumor cells that overexpress the CD44 receptor, HA has found extensive application as a targeting ligand for drug delivery systems.

### Chitosan

CS is a natural polysaccharide obtained from crustacean shells, which exhibits good biocompatibility and biodegradability [164]. Studies have shown that CS can induce bone formation and promote osteoblast growth in vivo, and it has been demonstrated that CS scaffolds can prolong drug release and encapsulate drugs for targeted delivery [165]. Thus, CS is an ideal vehicle for the sustained release of nanoparticles such as Exos. In one study, Bahar et al. [166] found that Exos-loaded CS/hydroxyapatite composites exhibited favorable bioactivity, induced angiogenesis, and wound healing, and increased bone mineral density in rat cranial defects. Additionally, Liu et al. [167] identified a novel 3D-printed collagen/CS scaffold loaded with insulin-like growth factor-1-pretreated NMSC Exos that enhanced repair and functional recovery after traumatic brain injury in rats.

### Polylactic acid

Polylactic acid (PLA) is a polyester obtained by the polymerization of lactic acid and is considered one of the most attractive polymer candidates for controlled drug delivery [168]. However, despite the mechanical stability and cytocompatibility advantages associated with PLA, further surface modifications are required to improve its bioactivity. In this context, PLA scaffolds modified with Exos have recently been used for bone repair. For example, Gandolfi et al. [169] used Exos-enriched PLA scaffolds to promote osteogenesis in human adipose MSCs, whereas Zhang et al. [170] demonstrated that bioactive 3D-printed PLA scaffolds modified with MSC Exos have immunomodulatory potential to favor osteogenic differentiation, indicating their promise for application in bone tissue regeneration. Additionally, Han et al. [171] constructed poly(aspartic acid)–poly(lactic acid)–hydroxyacetic acid copolymer Exos microcapsules with good stability, which promoted bone tendon healing in rotator cuff tears.

## The Delivery Approaches of Exos

### Hydrogels

Hydrogels are biocompatible and mechanically robust, resorbable biological platforms composed of 3D hydrophilic polymers that not only provide a suitable nutrient environment for the growth of endogenous cells but also exhibit injectable and adhesive properties [172]. This facilitates their maneuverability and promote the long-term attachment of endogenous cells to wounds during the healing process. The incorporation of Exos into hydrogels enhances their stability, maintains their biological activity, and represents a widely employed strategy for Exos delivery [173,174]. For example, Zhang et al. [175] fabricated a self-healing conductive hydrogel loaded with an Exos–metformin hybrid to improve wound healing by inhibiting mitochondrial fission. In addition, Yang et al. [176] constructed a gel system consisting of hUCMSC-Exos and Pluronic F-127 that promotes effective Exos delivery and enhances their therapeutic effects in diabetic wound treatment. In another study, Guan et al. [177] incorporated gelatin methacryloyl (GM) with aldehyde-functionalized chondroitin sulfate (OCS) to create GMOCS hydrogels, which were subsequently loaded with Exos. They found that the incorporation of OCS remarkably enhanced ECM synthesis within the GMOCS hydrogel. Moreover, the GMOCS-Exo-based hydrogel improved the adhesion of chondrocytes by reducing inflammation, leading to enhanced growth plate regeneration through remodeling of the ECM.

In recent years, the combination of Exos with other drugs for hydrogel loading has received increasing research attention [178]. In addition, self-healing hydrogels have gained attention for their ability to rebuild broken bonds after damage, thereby restoring their original mechanical, chemical, and electronic properties. Furthermore, as bacterial infection in the wound environment can prolong the healing process, it is necessary to develop hydrogel materials exhibiting both antimicrobial activity and self-healing properties. For example, Wang et al. used a simple chemical modification of HA and poly-ε-L-lysine to prepare hydrogels with self-healing ability via a Schiff base reaction and showed that the addition of Pluronic F127 imparted thermal responsiveness to the gels. Subsequently, AMSC Exos were loaded by electrostatic adsorption and used to promote chronic diabetic wound healing [179].

### Microspheres

Although Exos can be employed as effective agents in stem cell transplantation, their clinical use is limited by high clearance rates. It has therefore been considered that the encapsulation of Exos into hydrogels, which possess low viscosities and are easily injectable, may provide a promising approach for achieving sustained release. Nevertheless, the rapid and uncontrollable release rates of Exos from hydrogels fail to meet the requirements for continuous delivery. Although hydrogels with higher viscosities could potentially enhance Exos retention, these pose challenges for injection into the skin. As an alternative approach, microspheres have garnered vital interest due to their multiscale structures, functional properties, and injectable nature.

**Fig. 3.**
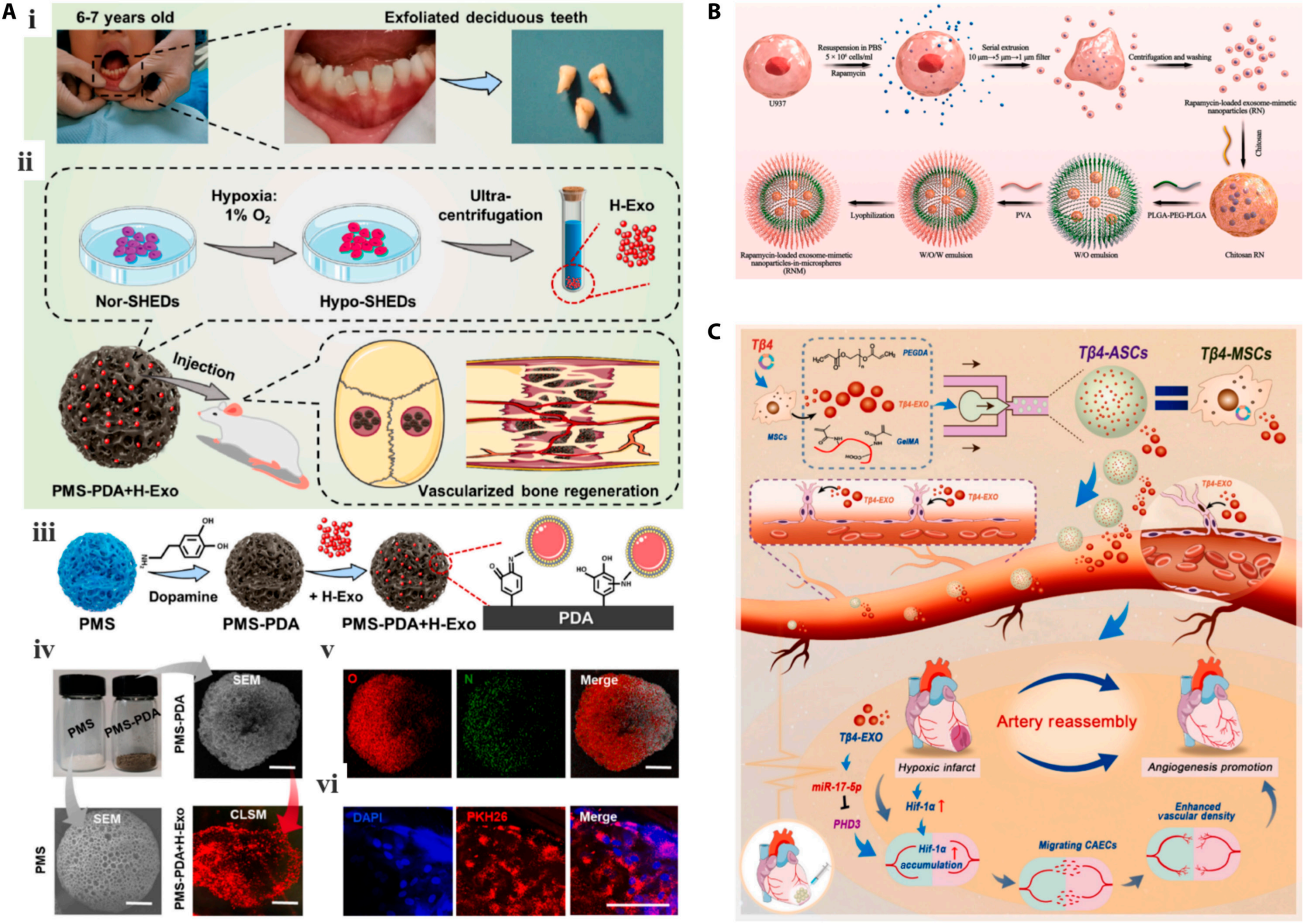
Microspheres for Exo delivery. (A) Exos-adsorbed PMS-PDA microsphere for the treatment of bone defects [182] (Copyright ©2019, Gao et al.). (B) Rapamycin-loaded Exos-mimetic nanoparticles PLGA microspheres preparation [183] (Copyright ©2019, Li et al.). (C) Tβ4-ASCs microspheres for myocardial infarction therapy [185] (Copyright ©2019, Chen et al.).

Microspheres are polymeric particle dispersion systems containing spherical or subspherical particles characterized by small sizes and high specific surface areas [180]. To date, they have been widely used as Exos carriers for controlled release, sustained release, and targeted delivery applications [174]. By adjusting the structures and compositions of microspheres, Exos release rates and characteristics can be modified [181]. For example, Gao et al. [182] prepared Exos-adsorbed poly(lactide-co-glycolide)-coated poly(dopamine) microspheres (PMS-PDA), which efficiently adsorbed Exos and allowed their sustained release for up to 21 d (Fig. 3A). Notably, these Exos retained high biological activity and promoted vascularized bone regeneration in rat skull defects measuring 5 mm. In addition, Li et al. [183] fabricated rapamycin-loaded Exos-mimetic nanoparticles (RNs) derived from the human macrophage cell line U937 using an extrusion technique. Subsequently, the RNs were encapsulated within polylactic-co-glycolic acid (PLGA) microspheres (RNMs) (Fig. 3B). Both the RN and RNM species were demonstrated to potently inhibit cell proliferation, induce ​substantial apoptosis, and effectively suppress angiogenic factor expression in hemangioma stem cells. In another study, Yang et al. developed an injectable hydrogel system composed of the matrix metalloproteinase-1-sensitive self-assembling peptide KLDL-MMP1, GelMA. Bone marrow MSC Exos were then encapsulated within the hydrogel microspheres [184], which exhibited an average particle size range of 50 to 70 μm, rendering them suitable for use in minimally invasive injections. This innovative approach holds great promise as an effective delivery platform for Exos to accelerate neovascularized bone healing. In another study, microfluidics was used to encapsulate specific Exos within microspheres, enabling the development of engineered stem cells capable of sustainably releasing Tβ4 Exos (Tβ4-ASCs) that promote collateral formation after myocardial infarction, offering a promising alternative to clinical hemotransfusion reconstruction (Fig. 3C) [185].

### Microneedles

**Fig. 4.**
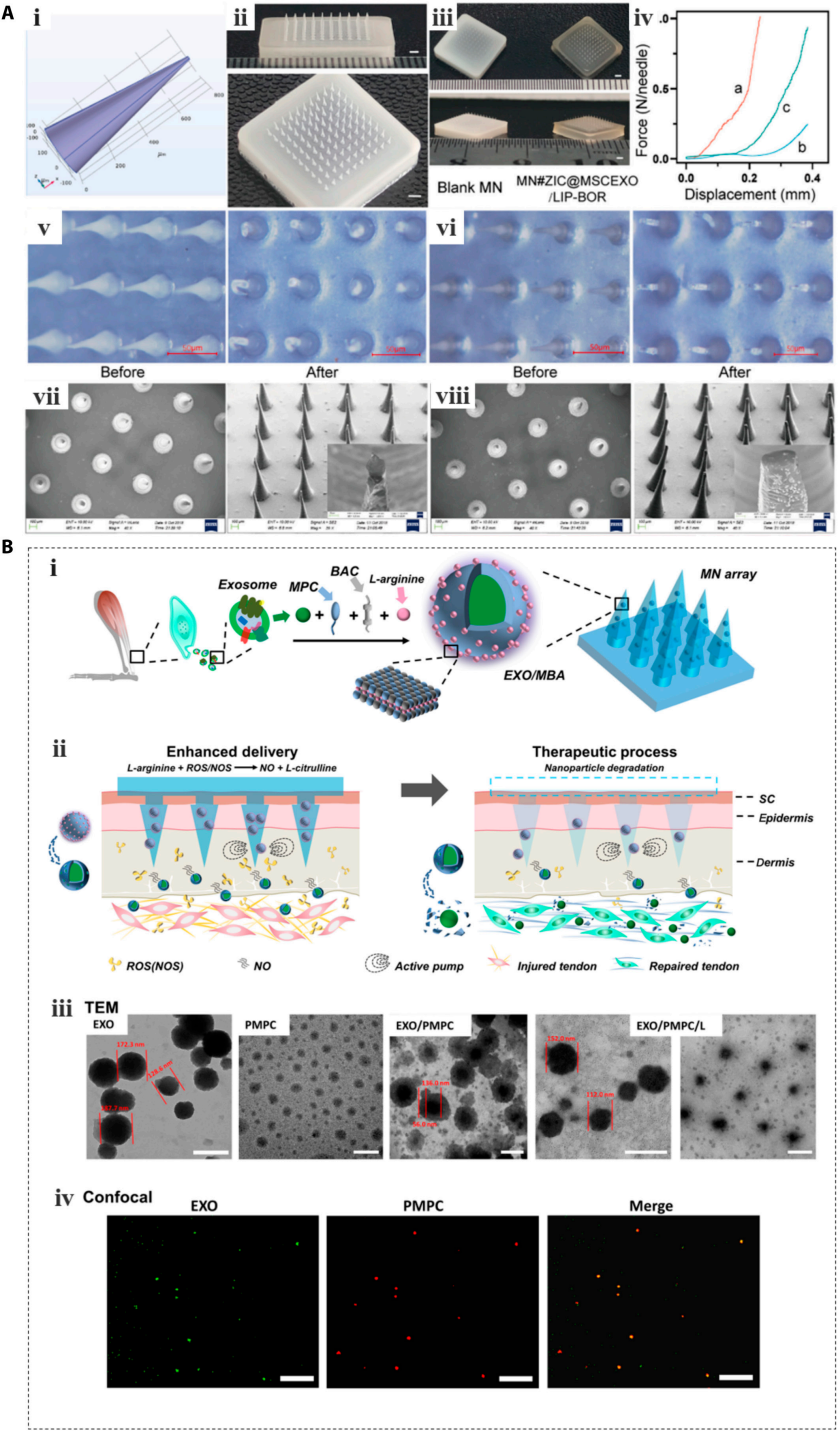
MNs for Exos delivery. (A) MN#ZIC@MSCEXO/LIP-BOR MN patches [187] (Copyright ©2023, published by Elsevier B.V.). (B) Schematic illustrations outlining the synthesis and verification of the EXO/MBA MN system [190] (Copyright ©2021, *ACS Nano*, published by the American Chemical Society).

Microneedles (MNs) are minimally invasive devices that painlessly penetrate the outer layer of the skin to facilitate the entry of large molecules. As MNs come into direct contact with the skin and the needle tips are partially inserted into the skin to achieve continuous drug release, the biodegradability and long-term biocompatibility of the MN material must be carefully optimized during the design process [186]. In this context, Song et al. [187] fused borneol-modified liposomes with Exos derived from MSCs and loaded ziconotide (ZIC) to prepare biocompatible MNs that enhanced the blood–brain barrier crossing efficiency of ZIC (Fig. 4A). In addition, Yang et al. developed a keratin-based MN transdermal delivery device for the codelivery of a hair follicle stem cell activator, MSC Exos, and the small-molecule drug UK5099. Using this system, follicular cycle transition was successfully modulated, and hair regeneration was promoted [188].

To enhance the therapeutic effectiveness of Exos, several innovative strategies have been proposed based on conventional MNs. For example, Zeng et al. [189] simultaneously inhibited inflammation and promoted angiogenesis at the wound site by incorporating M2 macrophage Exos in the needle tip and polydopamine nanoparticles in the backing layer. Notably, the use of mild photothermal therapy, facilitated by the photosensitive backing layer generating gentle heat (40 °C), contributed to enhanced angiogenesis and diabetic wound healing. Although MN arrays provide a noninvasive transdermal delivery system for Exos, passive diffusion limits the ability of Exos to fully reach the injury site, leading to reduced delivery efficiency. To address this, Liu et al. developed a detachable MN array to deliver nitric oxide nanomodified Exos for the treatment of Achilles tendinopathy. These nanomotors undergo a chemical reaction based on their surroundings (e.g., acid, H_2_O, or glucose), enabling their movement in the exhaust gases or solid waste (Fig. 4B) [190]. To alleviate the discomfort caused by substrate adhesion to the skin, indwelling MNs can be detached after application while preserving the needle tip within the tissue. In another study, Shi et al. [191] designed detachable MNs consisting of a dissolvable polyvinyl alcohol tip and a dissolvable HA matrix. Once inserted into the skin, the matrix was dissolved, whereas the dissolvable tip remained intact, permitting the continuous release of hair growth activators (Exos and L-lactate). These non-drug-dependent dissolvable MNs exhibit no side effects, ensuring good patient compliance with this treatment.

### 3D printing

ECM is essential for controlling cell proliferation, differentiation, and physiological functions. To replicate the natural characteristics of the ECM, it is crucial to develop tissue-engineered biomimetic scaffolds that can provide an appropriate microenvironment. Among the various approaches reported to date, the decellularized ECM (dECM) stands out for its ability to replicate the complexity of the native ECM [192]. However, traditional techniques, such as freeze-drying, fall short in terms of fully emulating the natural ECM structure. Thus, in recent years, 3D printing has emerged as a novel tool for precisely fabricating tissues or organs with intricate spatial architectures due to its high degree of freedom [193]. The exploitation of this technology for Exos delivery has gained ​notable attention for its ability to enhance therapeutic effects.

Exos delivery can be achieved via 3D printing by 2 main routes: encapsulation within a scaffold material or coating onto the scaffold itself. For example, using the 3D dECM technology, Li et al. [194] successfully regenerated cartilage and subchondral bone tissue by encapsulating Exos within a bionic hydrogel scaffold. In addition, Chen et al. [195] showcased the ability of an externally loaded 3D GelMA hydrogel to serve as a graft delivery system for Exos, enabling their noninvasive injection into injured lesions and inducing neurological recovery after SCI. Notably, the biocompatible 3D hydrogel-loaded Exos exhibited sustained release capabilities while promoting neural stem cell survival and neural differentiation in vitro. Furthermore, the implantation of the 3D hydrogel facilitated Tuj-1-positive neuron differentiation reduced astrocyte scarring and enhanced axonal growth, ultimately leading to neurological recovery post-SCI.

Despite these advances, the rapid degradation profiles of mono-material hydrogels hinder the process of tissue regeneration, and traditional photocrosslinking techniques pose challenges in terms of material patterning, particularly in 3D modeling. To address these issues, additional biomaterials, such as gelatin, Alg, collagen, and their derivatives, can be introduced to increase the applicability of the dECM hydrogel in 3D printing. For example, Chen et al. [195] combined MSC Exos, decellularized cartilage ECM, and a GelMA hydrogel as a bioink to fabricate 3D-printed scaffolds for osteochondral defect regeneration.

### Electrostatic spinning

Electrostatic spinning is a processing technique employed to fabricate ultrafine polymer fibers by subjecting a charged polymer solution to an electrostatic field beam. Electrospun fiber scaffolds, which exhibit structural similarity with the ECM, have become a prominent area of research interest. Exos can be incorporated into these electrospun fibers through surface adsorption, mixing, or emulsion methods. For example, Kang et al. [196] employed electrostatic spinning to fabricate a composite scaffold comprising PLGA and Mg-gallic acid metal–organic framework polymer co-hybrids, followed by the integration of Exos onto the composite scaffold. Subsequently, these functionalized scaffolds were implanted into rats for the treatment of cranial defects, and it was shown that the scaffolds promoted bone regeneration and angiogenesis, in addition to exhibiting anti-inflammatory effects. Phosphatidylserine (PS), a lipid present on the cell surface following axonal injury, has been demonstrated to play a key role in the detection and facilitation of axonal fusion and regeneration after injury. In this context, Su et al. [197] synthesized a PS aptamer, which was combined with extracted Exos via self-assembly techniques to construct PS-targeting Exos. Using electrostatic spinning technology, a bionic periosteum with a guided microstructure was fabricated to activate these Exos. The resulting bionic periosteum successfully enhanced neural axonal fusion, vascular regeneration, and bone regeneration. Consequently, this innovative bionic periosteum holds great promise for use as a therapeutic strategy for bone regeneration.

## Exos in Dermatologic Therapeutics

### Systemic lupus erythematosus

Systemic lupus erythematosus (SLE) affects multiple systems and organs and is characterized by an autoimmune-mediated pathology with diverse clinical manifestations, ranging from mild skin damage to severe multiorgan dysfunction [198]. Conventional treatments for SLE, such as immunomodulators and immunosuppressive drugs, have limited efficacy in controlling inflammation. Consequently, long-term immunosuppression is often required [199], which can lead to various adverse effects, including infections, secondary malignancies, and organ failure. Therefore, there is an urgent requirement for effective therapeutic options that offer sustained immunosuppressive effects with minimal side effects.

In recent years, Exos have been used for SLE early diagnosis and therapy. For example, Chuang et al. [200] identified a bactericidal/permeability-increasing protein (BPI), an exosomal protein from T cells of patients with SLE, and demonstrated that the overexpression of BPI in Exos plays a crucial role in triggering an autoimmune response. These findings suggest that Exos-containing BPI may have potential implications for the early diagnosis of SLE nephritis. In addition, Exos exert inhibitory effects on specific effector cells involved in the innate and adaptive immunities associated with SLE [45]. For example, MSC Exos maintain M2-type macrophage homeostasis by facilitating macrophage polarization toward anti-inflammatory phenotypes while also activating Treg cells [201]. Furthermore, MSC Exos suppress the immune responses mediated by the effector cells implicated in both innate and adaptive immunities while enhancing autoantibody-induced autoimmune reactions. In one study, Dou et al. [202] investigated the effects of MSC Exos on the M1-type polarization of macrophages and evaluated their underlying mechanism. They observed that MSC Exos suppressed the M1-type polarization of macrophages, leading to decreased levels of TNF-α and IL-1β. Conversely, they found that the removal of tsRNA-21109 from MSC Exos resulted in the up-regulation of M1 markers (CD 80, NOS 2, and MCP 1), down-regulation of M2 markers (CD 206, ARG 1, and MRC 2), and increased production of TNF-α and IL-1β in macrophages. These findings suggest that MSC Exos may inhibit macrophage M1-type polarization by transferring tsRNA-21109, thereby highlighting a possible therapeutic target for SLE. Additionally, Exos play an important role in recipient cells through miRNA-mediated mechanisms [203]. Among these miRNAs, miR-155 is highly conserved in mammals and has been implicated as a key regulator of cell proliferation in autoimmunity. Notably, miR-155 has been shown to regulate B cell activation during the immune response in SLE. In this context, Zhao et al. [204] demonstrated that Exos modulate B cell activation via the extracellular signal-regulated kinase signaling pathway by targeting miR-155 expression in B cells. This modulation effectively suppressed the persistent activation of autoreactive B cells and attenuated lupus-like disease progression.

### Atopic dermatitis

Atopic dermatitis (AD) is a chronic and highly sensitized form of skin inflammation characterized by the infiltration of immune cells [205]. The pathogenesis of AD is primarily associated with an enhanced Th2-mediated inflammatory response [206], where the impaired skin barrier function triggers inflammation. Although current therapeutic agents for AD (e.g., cortisol, calcineurin phosphatase inhibitors, and immunosuppressants) exhibit favorable efficacy against AD symptoms; their long-term use may be hindered by adverse effects. Therefore, effective and safe therapeutic strategies for AD are urgently needed.

Exos have been shown to attenuate AD-like symptoms by suppressing the expression levels of several inflammatory cytokines [207], and they also exhibit the potential for promoting skin regeneration [208]. For example, Cho et al. [209] demonstrated for the first time in an in vivo mouse model that ASC Exos improve AD by reducing the expression of various inflammatory cytokines. Yoon et al. used Exos secreted from IFN-γ-triggered multifunctional MSCs (iMSCs) to treat *Aspergillus fumigatus*-induced AD in mice. The results showed that IFN-γ-iExo improved the skin barrier function in AD mice, and this process was mediated by suppressing the T cell immune response [210].

### Psoriasis

The pathogenesis of psoriasis is closely linked to immune dysregulation, characterized by the overactivation of immune cells and interactions with keratinizing cells through inflammatory cytokines. This creates a vicious cycle within the cytokine network, resulting in progressive amplification of the inflammatory response at the lesion site, and ultimately leading to psoriasis [211]. Current therapeutic options for psoriasis are limited. In this context, Exos therapies have demonstrated promising outcomes in the treatment of psoriasis because of their exceptional biocompatibility, low cytotoxicity, and immunogenicity profiles, as well as their excellent target-specific nature.

MSCs have been widely studied due to their established safety record in human patients and their current approval for treating highly refractory inflammatory diseases. A study by Zhang et al. [212] showed that MSC Exos exert regulatory effects on psoriasis-like skin inflammation, in addition to attenuating imiquimod-induced psoriasis-like inflammation. It has also been demonstrated that neutrophil-derived vesicles are known to independently modulate the adaptive immune responses by targeting specific cell types. For example, Shao et al. [213] characterized the morphologies and compositions of neutrophil Exos while investigating their impact on generalized pustular psoriasis-related gene expression in keratinocytes. Their findings highlighted the involvement of neutrophil Exos in autoinflammatory responses associated with generalized pustular psoriasis.

**Fig. 5.**
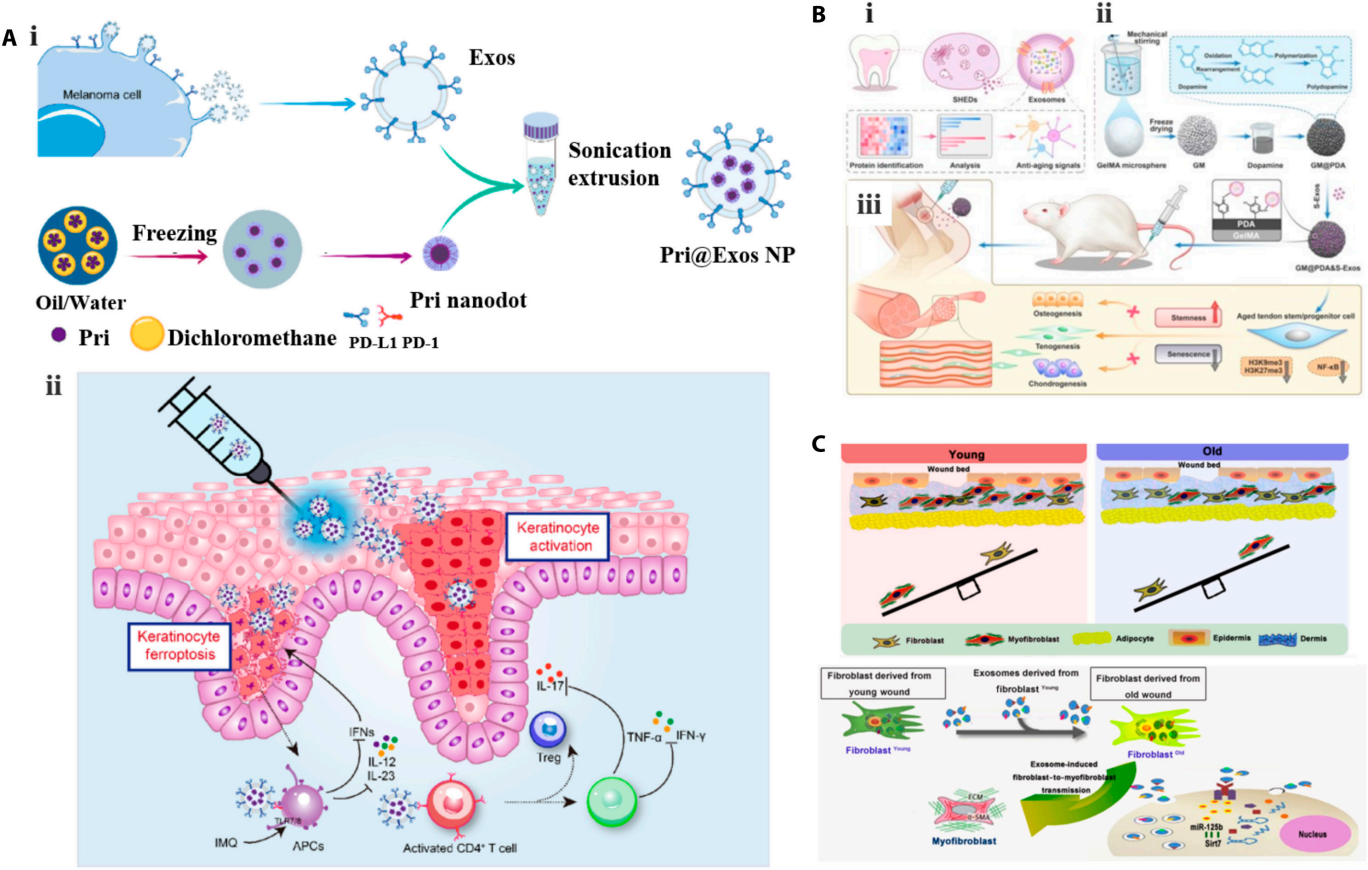
Exos for psoriasis and anti-aging therapy. (A) Diagram of the preparation and use of Pristimerin@Exos in the treatment of psoriasis [214] (Copyright ©2023, Jia et al.). (B) SHED Exos nanoparticles for aging-impaired tendon stem/progenitor cell therapy [219] (Copyright ©2023, *Advanced Materials*, published by Wiley-VCH GmbH). (C) Young fibroblasts Exos for wound healing in aged skin treatment [221] (Copyright ©2022, Xia et al.).

Recently, Exos have also been used as carriers for drug delivery. For example, Jia et al. [214] employed melanoma cell Exos for the targeted delivery of pristimerin (a naturally occurring triterpenoid bioactive compound that inhibits activated CD4^+^ T cells and keratinocytes) to overactive cells during psoriasis inflammation (Fig. 5A). It was found that the pristimerin/Exos system effectively targeted cutaneous psoriatic lesions in mice. Furthermore, the dysregulation of several miRNAs has been identified in psoriasis. In this context, Liu et al. [215] demonstrated the therapeutic potential of Exos in delivering miR-124-3p to keratinocytes to modulate cytokine expression, thereby offering novel insights into the treatment of psoriasis.

### Skin aging

With advancing age, the fibroblasts in the skin experience a decline in vitality and functionality, leading to reduced production of collagen, elastic fibers, and HA. Consequently, this results in dermal thinning accompanied by sagging and wrinkling of the skin. Henceforth, the diminished vitality and quantity of fibroblasts serve as the fundamental cause underlying skin aging [216]. The aging process is also influenced by damage to the ECM [217], such as oxidative stress, which induces ECM degradation and disintegration. At the same time, senescent and damaged cells cause inflammation and erythema during skin aging. Thus, to mitigate skin aging, research in the field of regenerative medicine has primarily focused on exploring novel antioxidants and immunomodulators, including the direct application of MSCs [218].

Exos have also been found to modulate histone methylation to reverse aged tendon stem/progenitor cells (AT-SC) aging (Fig. 5B) [219]. Furthermore, the antiaging role of Exos derived from various stem cell sources has been extensively studied [27]. For example, Hu et al. [220] used needle-free jet injection to administer 3D cultured human dermal fibroblast (HDF) Exos into nude mice exposed to ultraviolet B (UVB) irradiation (311 nm) and observed that these Exos effectively restored the function of the aged HDFs. They also demonstrated that the 3D HDF-EXO system could modulate dermal fibroblasts, promoting collagen biosynthesis and alleviating the skin inflammation induced by UVB irradiation. Xia et al. [221] found that the Exos miRNA-125b derived from young fibroblasts promotes myofibroblast differentiation and wound healing in aged mice (Fig. 5C). In addition, it is well known that adipose-derived stem cells (ADSCs) possess a self-renewal ability leading to a capacity to reduce the appearance of photoaging wrinkles and enhance collagen production in photoaging fibroblasts in vitro [222]. With this in mind, Guo et al. [223] compared the effects of ADSC Exos on the cell activity and morphology of skin fibroblasts cultured in vitro to investigate their antiaging potential. Compared with adipose stem cells alone, ADSC Exos offered various advantages, such as stable preservation capability, low tumorigenicity, and low immunogenicity, thereby representing a promising cell-free therapeutic approach for combating aging.

As an alternative, plant Exos are also extensively employed in the field of dermatology. Similar to animal Exos, plant Exos exhibit a comparable size distribution, surface charge, surface morphology, and content composition. These vesicles encapsulate a diverse array of molecules, including RNA, proteins, and lipids, which are known to govern various physiological processes [224]. In this context, Trentini et al. [225] investigated the effects of apple-derived nanovesicles (ADNVs) on skin aging and repair and found that the ADNVs improved type I collagen synthesis and reduced matrix metalloproteinase (MMP) production in primary dermal fibroblasts by attenuating Toll-like receptor 4 (TLR4)-induced signaling and down-regulating the NF-κB pathway. In addition, milk has been used as an effective nutrient in skin care and health. Recent research shows that milk Exos can be used to achieve skin antiaging in a number of dimensions, including barrier repair, promotion of natural moisturizing factor production, inhibition of collagen degradation, and promotion of trauma repair [226]. For example, Han et al. [227] showed that bovine colostrum Exos enhanced collagen production while concurrently reducing ROS and melanin synthesis in diverse skin cell types, thereby indicating the potential applicability of bovine milk Exos in advanced cell-free skin rejuvenation therapies.

### Wound healing

Poor wound healing is currently a major clinical problem and can be attributed to various factors, including insufficient angiogenesis, inflammation, and chronic hypoxia [228,229]. As the wound microenvironment is unfavorable for stem cell survival and function, the use of MSC Exos containing mRNA, miRNA, cytokines, and growth factors presents a novel cell-free approach to support wound healing and skin regeneration.

The role of Exos in promoting wound healing is mediated by their ability to modulate the inflammation, proliferation, and remodeling phases of wound healing [230]. During the inflammatory phase, Exos exhibit immunomodulatory capabilities similar to those of MSCs. They can influence various immune cells, such as inducing macrophages toward an anti-inflammatory M2 phenotype, inhibiting B cell maturation, suppressing T cell proliferation, and converting T cells into T regulatory cells. In addition, during the proliferative phase, Exos directly impact resident cell proliferation and differentiation while also promoting angiogenesis at the injury site [231]. Moreover, during the matrix remodeling phase, Exos facilitate collagen and elastin synthesis to minimize scar formation [232].

**Fig. 6.**
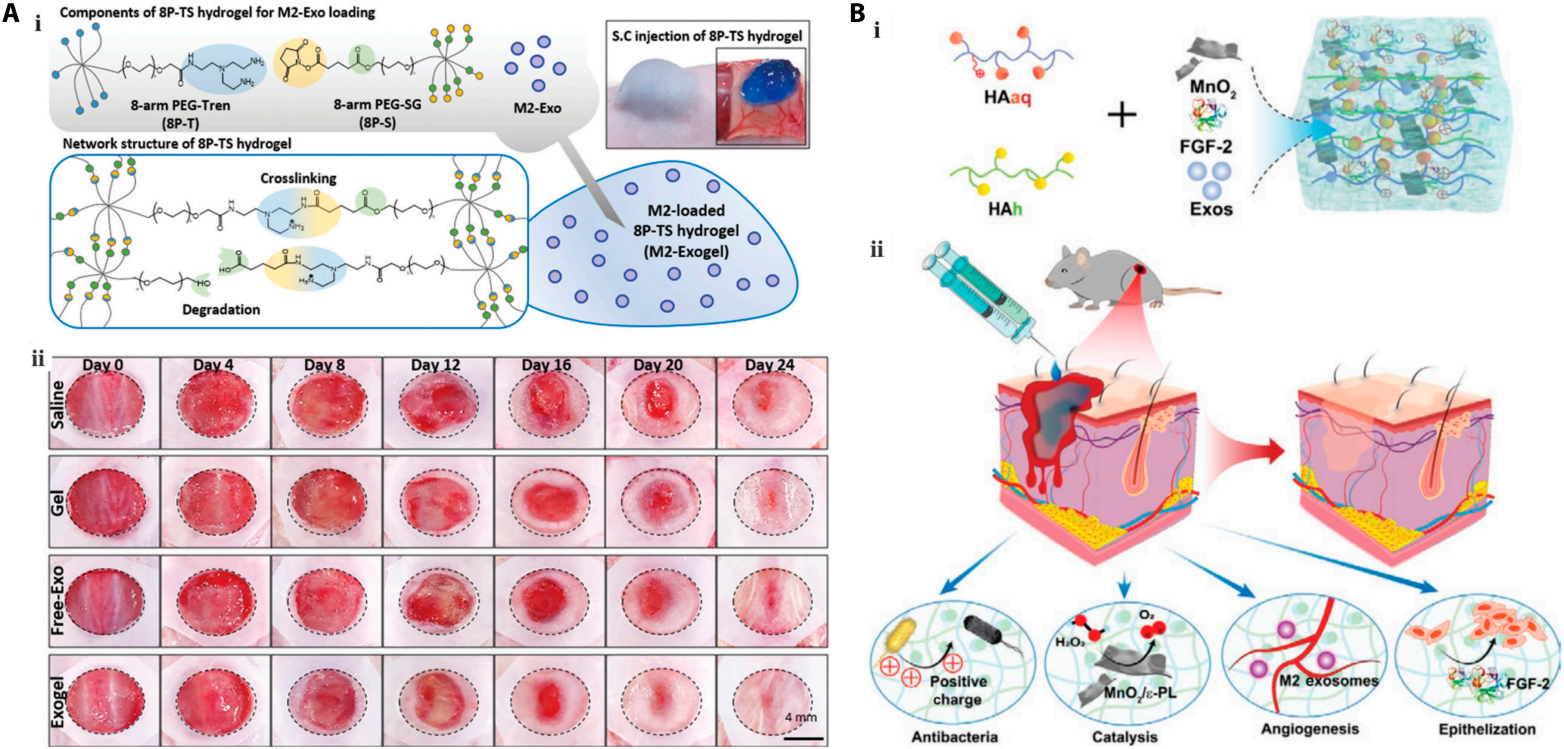
Exos for promoting skin wound healing. (A) Preparation of PEG-based injectables and promotion of wound healing hydrogels [234] (Copyright ©2021, *Small*, published by Wiley-VCH GmbH). (B) Synthesis of HA@MnO_2_/FGF-2/Exo hydrogel and mechanism of promoting wound healing [235] (Copyright ©2021, *Small*, published by Wiley-VCH GmbH).

The sustained release of Exos at the wound site often requires a carrier to function effectively, as Exos are rapidly cleared by the body upon application [233]. In this context, Yang et al. [176] used Pluronic F-127 for the retention and continuous release of hUCMSC Exos in damaged tissue, which promoted wound repair. The study found that at 14 days after wound modeling, wounds in the hUCMSC Exos/PF-127 group were almost completely healed, while the wound healing rates in the hUCMSC Exos group and the PF-127 hydrogel group were only 8.95% and 14.52%, respectively. This suggests that the effective delivery of hUCMSC Exos and improved Exo capacity in PF-127 hydrogel can promote diabetic wound healing. Furthermore, Kwak et al. utilized PEG hydrogels for the sustained release of Exos to guide macrophage polarization in skin wound healing (Fig. 6A). The results showed that wounds treated with Exogels demonstrated accelerated wound closure and enhanced healing quality by promoting the polarization of macrophages from M1 to M2 type [234].

Wound infection is also a major cause of delayed wound healing, and the synergistic effect of Exo and antimicrobial materials to promote wound healing is a novel idea. In this context, Xiong et al. [235] designed an injectable HA@MnO_2_/FGF-2/Exo hydrogel (Fig. 6B), in which MnO_2_ acts as an antimicrobial agent and FGF-2/Exos synergistically promote angiogenesis, attenuate inflammation, and reduce oxidative stress levels. This hydrogel demonstrated the ability to form a protective barrier that covers the wound, facilitating rapid hemostasis and providing long-term antimicrobial protection to enhance wound healing.

### Systemic sclerosis

Systemic sclerosis (SSc) is a group of systemic autoimmune diseases characterized by dermal fibrosis and tissue induration. The pathogenesis of SSc remains intricate and incompletely elucidated, posing challenges in terms of patient classification and treatment selection [236]. Numerous preclinical and clinical investigations are currently underway to explore the therapeutic potential of stem cells, particularly MSCs, for the treatment of SSc [237]. However, due to the large sizes of MSCs, it is challenging for them to access and undergo transplantation at the target site. The small EVs of stem cells have recently been demonstrated to modulate the immune system through multiple mechanisms [238].

Macrophages and fibroblasts were demonstrated to be involved in the crosstalk process in SSc skin, leading to mutual activation, inflammation, and ECM deposition. Regulation of the M1/M2 macrophage balance is, therefore, an effective strategy for the treatment of SSc. In addition, Yu et al. [239] demonstrated that hUCMSC Exos increased ECM deposition and inhibited the epithelial–mesenchymal transition process. Combined with their potential antifibrotic and anti-inflammatory effects, hUCMSC Exos could be considered a candidate therapy for SSc. Moreover, the miRNAs present in Exos have been demonstrated to play a pivotal role in fibrosis pathogenesis. By inducing the disruption or translational repression of specific mRNAs, miRNAs can exert precise control over gene expression and serve as crucial epigenetic regulators during the development of dermatofibrosis. In this context, Wang et al. [240] elucidated that ADMSC Exos attenuated fibrosis in restrictive scleroderma through modulation of the let-7a-5p/TGF-βR1/Smad axis. Xie et al. showed that Exos hold great potential for drug delivery to target skin fibrosis. More specifically, the miR-214 delivered by their developed BMSC Exo system mitigated skin fibrosis by inhibiting the IL-33/ST2 axis in both SSc cells and animal models [241].

## Summary and Outlook

As a current research hotspot, Exos have demonstrated their potential in various areas of tissue regeneration, including those related to the skin. Possessing a variety of effective properties for use in skin therapy, their angiogenic, collagen-synthesizing, and inflammation-modulating abilities enable Exos to overcome the limitations of current skin therapies. As a result, they are expected to play an important role in the field of aesthetics as key ingredients in skin care products. However, the high costs associated with Exos technologies have hindered their widespread adoption to date. Current methods for the extraction and purification of Exos tend to be expensive, low-yielding, and time-consuming, in addition to providing limited purity levels. Although recent studies have demonstrated that a strategic combination of multiple techniques can effectively address these challenges, further investigations are necessary to determine the optimal combination of techniques that can provide efficient and cost-effective extraction and purification methods. To overcome the low yields and expensive nature of Exos, cell-derived nanovesicles (CDNs) could be considered as an alternative. CDNs can be artificially synthesized using living cells and offer 100- to 250-fold higher yields compared to naturally secreted Exos. Although the therapeutic efficacies of CDNs are still under investigation, they exhibit similar biochemical, structural, and functional properties to natural Exos. The future application of CDNs in biomedicine is therefore expected to hold great promise. Despite the availability of numerous assays for characterizing Exos, there is a lack of standardized guidelines for assessing their purity. Discrepancies in the measurement results obtained from different methods can be attributed to variations in the minimum detection size associated with each technique. TEM enables the detection of even the smallest vesicles, whereas dedicated flow cytometry allows for the accurate and rapid analysis of the vesicle size. However, currently, there is no definitive gold standard available for Exos identification; therefore, a combination of multiple identification methods is necessary to achieve comprehensive characterization. In addition, current biological experiments involving Exos are primarily limited to mouse and rat models, necessitating additional animal model experiments to validate their therapeutic effects in other organisms. Moreover, validation studies investigating the therapeutic efficacies of Exos often include blank control experiments but fail to demonstrate their superiority over other therapeutic agents within their class. In addition to the heterogeneity of Exos, the biogenesis of Exos involves multiple steps regulated by various mechanisms. How these different regulatory mechanisms work together remains an open question. Moreover, the specific mechanisms that characterize most Exos have not yet been elucidated.

In conclusion, Exos have garnered attention in the field of regeneration as a novel cell-free approach. As potential biomarkers and therapeutic targets, the application of Exos in clinical diagnosis and therapy has attracted ​​notable attention. Particularly in the fields of tumor diagnosis, neurodegenerative diseases, and cardiovascular diseases, Exos show great potential for application. More clinical studies are needed in the future to verify the validity and reliability of Exos as diagnostic markers. Further investigations are warranted to elucidate the molecular mechanisms underlying stem cell Exos therapy, including their source, isolation methods, culture conditions, and drug delivery protocols. Moreover, expedited efforts toward achieving standardized mass production are anticipated. It is envisaged that in the future, stem cell Exos will not only revolutionize dermatology but also lead to remarkable breakthroughs in the treatment of various systemic diseases.

## References

[1] Li H, Zhang J, Tan M, Yin Y, Song Y, Zhao Y, Yan L, Li N, Zhang X, Bai J, et al. Exosomes based strategies for cardiovascular diseases: Opportunities and challenges. Biomaterials. 2024;308:122544. 38579591 10.1016/j.biomaterials.2024.122544

[2] Gong T, Liu Y-T, Fan J. Exosomal mediators in sepsis and inflammatory organ injury: Unraveling the role of exosomes in intercellular crosstalk and organ dysfunction. Mil Med Res. 2024;11(1):24. 38644472 10.1186/s40779-024-00527-6 PMC11034107

[3] Yang Q, Li S, Ou H, Zhang Y, Zhu G, Li S, Lei L. Exosome-based delivery strategies for tumor therapy: An update on modification, loading, and clinical application. J Nanobiotechnology. 2024;22(1):41. 38281957 10.1186/s12951-024-02298-7 PMC10823703

[4] Pan W, Chen H, Wang A, Wang F, Zhang X. Challenges and strategies: Scalable and efficient production of mesenchymal stem cells-derived exosomes for cell-free therapy. Life Sci. 2023;319: Article 121524. 36828131 10.1016/j.lfs.2023.121524

[5] Huang S, Ji X, Jackson KK, Lubman DM, Ard MB, Bruce TF, Marcus RK. Rapid separation of blood plasma exosomes from low-density lipoproteins via a hydrophobic interaction chromatography method on a polyester capillary-channeled polymer fiber phase. Anal Chim Acta. 2021;1167: Article 338578. 34049630 10.1016/j.aca.2021.338578 PMC8164660

[6] Lozano-Andrés E, Enciso-Martinez A, Gijsbers A, Ridolfi A, Van Niel G, Libregts SF, Pinheiro C, van Herwijnen MJ, Hendrix A, Brucale M. Physical association of low density lipoprotein particles and extracellular vesicles unveiled by single particle analysis. J Extracell Vesicles. 2023;12(11):12376. 37942918 10.1002/jev2.12376 PMC10634195

[7] Altıntaş O, Saylan Y. Exploring the versatility of exosomes: A review on isolation, characterization, detection methods, and diverse applications. Anal Chem. 2023;95(44):16029–16048. 37874907 10.1021/acs.analchem.3c02224

[8] Kumar K, Kim E, Alhammadi M, Reddicherla U, Aliya S, Tiwari JN, Park HS, Choi JH, Son CY, Vilian AE, et al. Recent advances in microfluidic approaches for the isolation and detection of exosomes. TrAC Trends Anal Chem. 2023;159: Article 116912.

[9] Lai JJ, Chau ZL, Chen SY, Hill JJ, Korpany KV, Liang NW, Lin LH, Lin YH, Liu JK, Liu YC, et al. Exosome processing and characterization approaches for research and technology development. Adv Sci. 2022;9(15):2103222. 10.1002/advs.202103222 PMC9130923 35332686

[10] Koksal AR, Ekmen N, Aydin Y, Nunez K, Sandow T, Delk M, Moehlen M, Thevenot P, Cohen A, Dash S. A single-step immunocapture assay to quantify HCC exosomes using the highly sensitive fluorescence nanoparticle-tracking analysis. J Hepatocell Carcinoma. 2023;10:1935–1954. 37936599 10.2147/JHC.S423043 PMC10627088

[11] Steć A, Jońca J, Waleron K, Waleron M, Płoska A, Kalinowski L, Wielgomas B, Dziomba S. Quality control of bacterial extracellular vesicles with total protein content assay, nanoparticles tracking analysis, and capillary electrophoresis. Int J Mol Sci. 2022;23(8):4347. 35457164 10.3390/ijms23084347 PMC9028362

[12] Wang C, Jin D, Yu Y, Tang L, Sun Y, Sun Z, Zhang G-J. A dual antibody-modified nanochannel biosensor for capture and identification of exosomes. Sensors Actuators B Chem. 2020;314: Article 128056.

[13] Alexandre L, Shen ML, de Araujo LOF, Renault J, DeCorwin-Martin P, Martel R, Ng A, Juncker D. Effect of sample preprocessing and size-based extraction methods on the physical and molecular profiles of extracellular vesicles. ACS Sens. 2024;9(3):1239–1251. 38436286 10.1021/acssensors.3c02070 PMC10964911

[14] Jafari D, Shajari S, Jafari R, Mardi N, Gomari H, Ganji F, Forouzandeh Moghadam M, Samadikuchaksaraei A. Designer exosomes: A new platform for biotechnology therapeutics. BioDrugs. 2020;34(5):567–586. 32754790 10.1007/s40259-020-00434-x PMC7402079

[15] Uddin N, Binzel DW, Shu D, Fu T-M, Guo P. Targeted delivery of RNAi to cancer cells using RNA-ligand displaying exosome. Acta Pharm Sin B. 2023;13(4):1383–1399. 37139430 10.1016/j.apsb.2022.11.019 PMC10149909

[16] García-Fernández J, de la Fuente Freire M. Exosome-like systems: Nanotechnology to overcome challenges for targeted cancer therapies. Cancer Lett. 2023;561: Article 216151. 37001751 10.1016/j.canlet.2023.216151

[17] Lu M, Huang Y. Bioinspired exosome-like therapeutics and delivery nanoplatforms. Biomaterials. 2020;242: Article 119925. 32151860 10.1016/j.biomaterials.2020.119925

[18] Yu H, Feng H, Zeng H, Wu Y, Zhang Q, Yu J, Hou K, Wu M. Exosomes: The emerging mechanisms and potential clinical applications in dermatology. Int J Biol Sci. 2024;20(5):1778. 38481799 10.7150/ijbs.92897 PMC10929203

[19] Zhang R, Wei Y, Wang T, Nie X, Shi Z, Deng Y, Li D. Exosomal miRNAs in autoimmune skin diseases. Front Immunol. 2023;14:1307455. 38106405 10.3389/fimmu.2023.1307455 PMC10722155

[20] Shang Y, Li M, Zhang L, Han C, Shen K, Wang K, Li Y, Zhang Y, Luo L, Jia Y, et al. Exosomes derived from mouse vibrissa dermal papilla cells promote hair follicle regeneration during wound healing by activating Wnt/β-catenin signaling pathway. J Nanobiotechnol. 2024;22(1):425. 10.1186/s12951-024-02689-w PMC11264511 39030543

[21] Dan X, Li S, Chen H, Xue P, Liu B, Ju Y, Lei L, Li Y, Fan X. Tailoring biomaterials for skin anti-aging. Mater Today Bio. 2024;28: Article 101210. 10.1016/j.mtbio.2024.101210 PMC11402947 39285945

[22] Yang P, Ju Y, Shen N, Zhu S, He J, Yang L, Lei J, He X, Shao W, Lei L, et al. Exos-loaded Gox-modified smart-response self-healing hydrogel improves the microenvironment and promotes wound healing in diabetic wounds. Adv Healthc Mater. 14(7):e2403304. 10.1002/adhm.202403304 39473310

[23] Tang Y, Kang Y, Zhang X, Cheng C. Mesenchymal stem cell exosomes as nanotherapeutics for dry age-related macular degeneration. J Control Release. 2023;357:356–370. 37028452 10.1016/j.jconrel.2023.04.003

[24] Zou J, Yang W, Cui W, Li C, Ma C, Ji X, Hong J, Qu Z, Chen J, Liu A, et al. Therapeutic potential and mechanisms of mesenchymal stem cell-derived exosomes as bioactive materials in tendon–bone healing. J Nanobiotechnology. 2023;21(1):14. 36642728 10.1186/s12951-023-01778-6 PMC9841717

[25] Wang T, Jian Z, Baskys A, Yang J, Li J, Guo H, Hei Y, Xian P, He Z, Li Z, et al. MSC-derived exosomes protect against oxidative stress-induced skin injury via adaptive regulation of the NRF2 defense system. Biomaterials. 2020;257: Article 120264. 32791387 10.1016/j.biomaterials.2020.120264

[26] Bian D, Wu Y, Song G, Azizi R, Zamani A. The application of mesenchymal stromal cells (MSCs) and their derivative exosome in skin wound healing: A comprehensive review. Stem Cell Res Ther. 2022;13(1):24. 35073970 10.1186/s13287-021-02697-9 PMC8785459

[27] Najafabadi AH, Soheilifar MH, Masoudi-Khoram N. Exosomes in skin photoaging: Biological functions and therapeutic opportunity. Cell Commun Signal. 2024;22(1):32. 38217034 10.1186/s12964-023-01451-3 PMC10785444

[28] Xiao Y, Li H, Zhang J, Yang S, Zhang C, Huang Y, Tang X, Xie H. Mesenchymal stem cell-derived exosomes: Versatile nanomaterials for skin wound treatment. Nano Res. 2024;17(4):2836–2856.

[29] Thakur A, Parra DC, Motallebnejad P, Brocchi M, Chen HJ. Exosomes: Small vesicles with big roles in cancer, vaccine development, and therapeutics. Bioactive Mater. 2022;10:281–294. 10.1016/j.bioactmat.2021.08.029 PMC8636666 34901546

[30] Zhang J, Zhu Y, Guan M, Liu Y, Lv M, Zhang C, Zhang H, Zhang Z. Isolation of circulating exosomes and identification of exosomal PD-L1 for predicting immunotherapy response. Nanoscale. 2022;14(25):8995–9003. 35700522 10.1039/d2nr00829g

[31] Gurung S, Perocheau D, Touramanidou L, Baruteau J. The exosome journey: From biogenesis to uptake and intracellular signalling. Cell Commun Signal. 2021;19(1):47. 33892745 10.1186/s12964-021-00730-1 PMC8063428

[32] Kalluri R, LeBleu VS. The biology, function, and biomedical applications of exosomes. Science. 2020;367(6478):eaau6977. 32029601 10.1126/science.aau6977 PMC7717626

[33] Zhao Y, Liu L, Sun R, Cui G, Guo S, Han S, Li Z, Bai T, Teng L. Exosomes in cancer immunoediting and immunotherapy. Asian J Pharm Sci. 2022;17(2):193–205. 35582642 10.1016/j.ajps.2021.12.001 PMC9091780

[34] Nila IS, Sumsuzzman DM, Khan ZA, Jung JH, Kazema AS, Kim SJ, Hong Y. Identification of exosomal biomarkers and its optimal isolation and detection method for the diagnosis of Parkinson’s disease: A systematic review and meta-analysis. Ageing Res Rev. 2022;82: Article 101764. 36273807 10.1016/j.arr.2022.101764

[35] Preethi KA, Selvakumar SC, Ross K, Jayaraman S, Tusubira D, Sekar D. Liquid biopsy: Exosomal microRNAs as novel diagnostic and prognostic biomarkers in cancer. Mol Cancer. 2022;21(1):54. 35172817 10.1186/s12943-022-01525-9 PMC8848669

[36] Cai S, Pataillot-Meakin T, Shibakawa A, Ren R, Bevan CL, Ladame S, Ivanov AP, Edel JB. Single-molecule amplification-free multiplexed detection of circulating microRNA cancer biomarkers from serum. Nat Commun. 2021;12(1):3515. 34112774 10.1038/s41467-021-23497-y PMC8192752

[37] Kim SB. Function and therapeutic development of exosomes for cancer therapy. Arch Pharm Res. 2022;45(5):295–308. 35604532 10.1007/s12272-022-01387-1 PMC9125016

[38] Fu H, Chen Y, Fu Q, Lv Q, Zhang J, Yang Y, Tan P, Wang X, Yang Y, Wu Z. From conventional to cutting-edge: Exosomes revolutionizing nano-drug delivery systems. Chem Eng J. 2024;500: Article 156685.

[39] Song F, Wang C, Wang C, Wang J, Wu Y, Wang Y, Liu H, Zhang Y, Han L. Multi-phenotypic exosome secretion profiling microfluidic platform for exploring single-cell heterogeneity. Small Methods. 2022;6(9):2200717. 10.1002/smtd.202200717 35901289

[40] Ding L, Yang X, Gao Z, Effah CY, Zhang X, Wu Y, Qu L. A holistic review of the state-of-the-art microfluidics for exosome separation: An overview of the current status, existing obstacles, and future outlook. Small. 2021;17(29):2007174. 10.1002/smll.202007174 34047052

[41] Almeria C, Kreß S, Weber V, Egger D, Kasper C. Heterogeneity of mesenchymal stem cell-derived extracellular vesicles is highly impacted by the tissue/cell source and culture conditions. Cell Biosci. 2022;12(1):51. 35501833 10.1186/s13578-022-00786-7 PMC9063275

[42] Morales R-TT, Ko J. Future of digital assays to resolve clinical heterogeneity of single extracellular vesicles. ACS Nano. 2022;16(8):11619–11645. 35904433 10.1021/acsnano.2c04337 PMC10174080

[43] Vermeer PD. Exosomal induction of tumor innervation. Cancer Res. 2019;79(14):3529–3535. 31088834 10.1158/0008-5472.CAN-18-3995 PMC6635078

[44] Zhao N, Deng Q, Zhu C, Zhang B. Application of extracellular vesicles in aquatic animals: A review of the latest decade. Rev Fisher Sci Aquacult. 2022;30(4):447–466.

[45] Zhao Z, Zhang L, Ocansey DKW, Wang B, Mao F. The role of mesenchymal stem cell-derived exosome in epigenetic modifications in inflammatory diseases. Front Immunol. 2023;14:1166536. 37261347 10.3389/fimmu.2023.1166536 PMC10227589

[46] Wang S, Lei B, Zhang E, Gong P, Gu J, He L, Han L, Yuan Z. Targeted therapy for inflammatory diseases with mesenchymal stem cells and their derived exosomes: From basic to clinics. Int J Nanomedicine. 2022;17:1757–1781. 35469174 10.2147/IJN.S355366 PMC9034888

[47] Abbaszadeh S, Nosrati-Siahmazgi V, Musaie K, Rezaei S, Qahremani M, Xiao B, Santos HA, Shahbazi M-A. Emerging strategies to bypass transplant rejection via biomaterial-assisted immunoengineering: Insights from islets and beyond. Adv Drug Deliv Rev. 2023;200:115050. 37549847 10.1016/j.addr.2023.115050

[48] Lotfy A, AboQuella NM, Wang H. Mesenchymal stromal/stem cell (MSC)-derived exosomes in clinical trials. Stem Cell Res Ther. 2023;14(1):66. 37024925 10.1186/s13287-023-03287-7 PMC10079493

[49] Kou M, Huang L, Yang J, Chiang Z, Chen S, Liu J, Guo L, Zhang X, Zhou X, Xu X. Mesenchymal stem cell-derived extracellular vesicles for immunomodulation and regeneration: A next generation therapeutic tool? Cell Death Dis. 2022;13(7):580. 35787632 10.1038/s41419-022-05034-x PMC9252569

[50] Guo M, Yin Z, Chen F, Lei P. Mesenchymal stem cell-derived exosome: A promising alternative in the therapy of Alzheimer’s disease. Alzheimers Res Ther. 2020;12:1–14. 10.1186/s13195-020-00670-x PMC7488700 32928293

[51] Qiu B, Xu X, Yi P, Hao Y. Curcumin reinforces MSC-derived exosomes in attenuating osteoarthritis via modulating the miR-124/NF-kB and miR-143/ROCK1/TLR9 signalling pathways. J Cell Mol Med. 2020;24(18):10855–10865. 32776418 10.1111/jcmm.15714 PMC7521270

[52] Gurunathan S, Kang M-H, Jeyaraj M, Qasim M, Kim J-H. Review of the isolation, characterization, biological function, and multifarious therapeutic approaches of exosomes. Cells. 2019;8(4):307. 30987213 10.3390/cells8040307 PMC6523673

[53] Nouri Z, Barfar A, Perseh S, Motasadizadeh H, Maghsoudian S, Fatahi Y, Nouri K, Yektakasmaei MP, Dinarvand R, Atyabi F. Exosomes as therapeutic and drug delivery vehicle for neurodegenerative diseases. J Nanobiotechnology. 2024;22(1):463. 39095888 10.1186/s12951-024-02681-4 PMC11297769

[54] Wang YL, Lin QS, Zhang H, Wang SC, Cui J, Hu Y, Liu JL, Li MM, Zhang K, Zhou FJ, et al. M2 macrophage-derived exosomes promote diabetic fracture healing by acting as an immunomodulator. Bioact Mater. 2023;28:273–283. 37303851 10.1016/j.bioactmat.2023.05.018 PMC10247878

[55] Huang LM, Wang F, Wang XP, Su CY, Wu SC, Yang C, Luo M, Zhang JY, Fu LW. M2-like macrophage-derived exosomes facilitate metastasis in non-small-cell lung cancer by delivering integrin αVβ3. MedComm. 2023;4(1):e191. 36582304 10.1002/mco2.191 PMC9789322

[56] Liu SJ, Chen J, Shi J, Zhou WY, Wang L, Fang WL, Zhong Y, Chen XH, Chen YF, Sabri A, et al. M1-like macrophage-derived exosomes suppress angiogenesis and exacerbate cardiac dysfunction in a myocardial infarction microenvironment. Basic Res Cardiol. 2020;115(2):22. 32112145 10.1007/s00395-020-0781-7

[57] Ji T, Zhao Y, Ding Y, Nie G. Using functional nanomaterials to target and regulate the tumor microenvironment: Diagnostic and therapeutic applications. Adv Mater. 2013;25(26):3508–3525. 23703805 10.1002/adma.201300299

[58] Hosseini R, Sarvnaz H, Arabpour M, Ramshe SM, Asef-Kabiri L, Yousefi H, Akbari ME, Eskandari N. Cancer exosomes and natural killer cells dysfunction: Biological roles, clinical significance and implications for immunotherapy. Mol Cancer. 2022;21(1):15. 35031075 10.1186/s12943-021-01492-7 PMC8759167

[59] Tang Q, Yang S, He G, Zheng H, Zhang S, Liu J, Wei S, Fan Q, Peng X, Li X, et al. Tumor-derived exosomes in the cancer immune microenvironment and cancer immunotherapy. Cancer Lett. 2022;548:215823. 35835409 10.1016/j.canlet.2022.215823

[60] Shang A, Gu C, Wang W, Wang X, Sun J, Zeng B, Chen C, Chang W, Ping Y, Ji P, et al. Exosomal circPACRGL promotes progression of colorectal cancer via the miR-142-3p/miR-506-3p-TGF-β1 axis. Mol Cancer. 2020;19(1):117. 32713345 10.1186/s12943-020-01235-0 PMC7384220

[61] Zhang W, Zheng X, Yu Y, Zheng L, Lan J, Wu Y, Liu H, Zhao A, Huang H, Chen W. Renal cell carcinoma-derived exosomes deliver lncARSR to induce macrophage polarization and promote tumor progression via STAT3 pathway. Int J Biol Sci. 2022;18(8):3209–3222. 35637970 10.7150/ijbs.70289 PMC9134902

[62] Baig MS, Roy A, Rajpoot S, Liu D, Savai R, Banerjee S, Kawada M, Faisal SM, Saluja R, Saqib U, et al. Tumor-derived exosomes in the regulation of macrophage polarization. Inflamm Res. 2020;69(5):435–451. 32162012 10.1007/s00011-020-01318-0

[63] Gunassekaran GR, Poongkavithai Vadevoo SM, Baek MC, Lee B. M1 macrophage exosomes engineered to foster M1 polarization and target the IL-4 receptor inhibit tumor growth by reprogramming tumor-associated macrophages into M1-like macrophages. Biomaterials. 2021;278: Article 121137. 34560422 10.1016/j.biomaterials.2021.121137

[64] Ahmadi M, Rezaie J. Tumor cells derived-exosomes as angiogenenic agents: Possible therapeutic implications. J Transl Med. 2020;18(1):249. 32571337 10.1186/s12967-020-02426-5 PMC7310379

[65] Zhao B, Lin H, Jiang X, Li W, Gao Y, Li M, Yu Y, Chen N, Gao J. Exosome-like nanoparticles derived from fruits, vegetables, and herbs: Innovative strategies of therapeutic and drug delivery. Theranostics. 2024;14(12):4598–4621. 39239509 10.7150/thno.97096 PMC11373634

[66] Cao M, Diao N, Cai X, Chen X, Xiao Y, Guo C, Chen D, Zhang X. Plant exosome nanovesicles (PENs): Green delivery platforms. Mater Horiz. 2023;10(10):3879–3894. 37671650 10.1039/d3mh01030a

[67] Pan Q, Bao Z, Wang Y, Wan T. RETRACTED: Nrf2 pathway activation with natural plant-derived exosome-like nanovesicle/hydrogel preparations for oxidative stress modulation in inflammation related diseases. Chem Eng J. 2024;480: Article 148282.

[68] Colombo M, Raposo G, Théry C. Biogenesis, secretion, and intercellular interactions of exosomes and other extracellular vesicles. Annu Rev Cell Dev Biol. 2014;30(1):255–289. 25288114 10.1146/annurev-cellbio-101512-122326

[69] Spada S. Study of microRNAs carried by exosomes, monitoring vesicular trafficking in cellular responses to stress. 2021;165(Part B):187–197. 10.1016/bs.mcb.2021.02.006 34311867

[70] Meldolesi J. Exosomes and ectosomes in intercellular communication. Curr Biol. 2018;28(8):R435–R444. 29689228 10.1016/j.cub.2018.01.059

[71] Isaac R, Reis FCG, Ying W, Olefsky JM. Exosomes as mediators of intercellular crosstalk in metabolism. Cell Metab. 2021;33(9):1744–1762. 34496230 10.1016/j.cmet.2021.08.006 PMC8428804

[72] van Niel G, Carter DRF, Clayton A, Lambert DW, Raposo G, Vader P. Challenges and directions in studying cell–cell communication by extracellular vesicles. Nat Rev Mol Cell Biol. 2022;23(5):369–382. 35260831 10.1038/s41580-022-00460-3

[73] Chang J, Zhang L, Li Z, Qian C, Du J. Exosomal non-coding RNAs(ncRNAs) as potential biomarkers in tumor early diagnosis. Biochim Biophys Acta Rev Cancer. 2024;1879(6): Article 189188. 39313040 10.1016/j.bbcan.2024.189188

[74] Xu Z, Zeng S, Gong Z, Yan Y. Exosome-based immunotherapy: A promising approach for cancer treatment. Mol Cancer. 2020;19(1):160. 33183286 10.1186/s12943-020-01278-3 PMC7661275

[75] Zhang L, Yu D. Exosomes in cancer development, metastasis, and immunity. Biochim Biophys Acta Rev Cancer. 2019;1871(2):455–468. 31047959 10.1016/j.bbcan.2019.04.004 PMC6542596

[76] Yao Y, Chen R, Wang G, Zhang Y, Liu F. Exosomes derived from mesenchymal stem cells reverse EMT via TGF-β1/Smad pathway and promote repair of damaged endometrium. Stem Cell Res Ther. 2019;10(1):225. 31358049 10.1186/s13287-019-1332-8 PMC6664513

[77] Singla D, Johnson T, Tavakoli Dargani Z. Exosome treatment enhances anti-inflammatory M2 macrophages and reduces inflammation-induced pyroptosis in doxorubicin-induced cardiomyopathy. Cells. 2019;8(10):1124. 31600901 10.3390/cells8101224 PMC6830113

[78] Huang Y, Yang L. Mesenchymal stem cell-derived extracellular vesicles in therapy against fibrotic diseases. Stem Cell Res Ther. 2021;12(1):435. 34348793 10.1186/s13287-021-02524-1 PMC8334330

[79] Xie Q, Zheng J, Ding J, Wu Y, Liu L, Yu Z, Chen G. Exosome-mediated immunosuppression in tumor microenvironments. Cells. 2022;11(12):1946. 35741075 10.3390/cells11121946 PMC9221707

[80] Del Vecchio F, Martinez Rodriguez V, Schukking M, Cocks A, Broseghini E, Fabbri M. Professional killers: The role of extracellular vesicles in the reciprocal interactions between natural killer, CD8^+^ cytotoxic T-cells and tumour cells. J Extracell Vesicles. 2021;10(6):e12075. 33815694 10.1002/jev2.12075 PMC8015281

[81] Vu NB, Nguyen HT, Palumbo R, Pellicano R, Fagoonee S, Pham PV. Stem cell-derived exosomes for wound healing: Current status and promising directions. Minerva Med. 2021;112(3):384–400. 33263376 10.23736/S0026-4806.20.07205-5

[82] Yu M, Liu W, Li J, Lu J, Lu H, Jia W, Liu F. Exosomes derived from atorvastatin-pretreated MSC accelerate diabetic wound repair by enhancing angiogenesis via AKT/eNOS pathway. Stem Cell Res Ther. 2020;11(1):350. 32787917 10.1186/s13287-020-01824-2 PMC7425015

[83] Hu Y, Rao SS, Wang ZX, Cao J, Tan YJ, Luo J, Li HM, Zhang WS, Chen CY, Xie H. Exosomes from human umbilical cord blood accelerate cutaneous wound healing through miR-21-3p-mediated promotion of angiogenesis and fibroblast function. Theranostics. 2018;8(1):169–184. 29290800 10.7150/thno.21234 PMC5743467

[84] Hu N, Cai Z, Jiang X, Wang C, Tang T, Xu T, Chen H, Li X, Du X, Cui W. Hypoxia-pretreated ADSC-derived exosome-embedded hydrogels promote angiogenesis and accelerate diabetic wound healing. Acta Biomater. 2023;157:175–186. 36503078 10.1016/j.actbio.2022.11.057

[85] Li D, Wu N. Mechanism and application of exosomes in the wound healing process in diabetes mellitus. Diabetes Res Clin Pract. 2022;187: Article 109882. 35487341 10.1016/j.diabres.2022.109882

[86] Baruah J, Wary KK. Exosomes in the regulation of vascular endothelial cell regeneration. Front Cell Dev Biol. 2020;7:353. 31998716 10.3389/fcell.2019.00353 PMC6962177

[87] Oveili E, Vafaei S, Bazavar H, Eslami Y, Mamaghanizadeh E, Yasamineh S, Gholizadeh O. The potential use of mesenchymal stem cells-derived exosomes as microRNAs delivery systems in different diseases. Cell Commun Signal. 2023;21(1):20. 36690996 10.1186/s12964-022-01017-9 PMC9869323

[88] Choi B, Lee C, Yu J-W. Distinctive role of inflammation in tissue repair and regeneration. Arch Pharm Res. 2023;46(2):78–89. 36719600 10.1007/s12272-023-01428-3

[89] Chang T, Wu C, Chiou S, Chang C, Liao H. Adipose-derived stem cell exosomes as a novel anti-inflammatory agent and the current therapeutic targets for rheumatoid arthritis. Biomedicines. 2022;10(7):1725. 35885030 10.3390/biomedicines10071725 PMC9312519

[90] Kim H, Wang SY, Kwak G, Yang Y, Kwon IC, Kim SH. Exosome-guided phenotypic switch of M1 to M2 macrophages for cutaneous wound healing. Adv Sci. 2019;6(20):1900513. 10.1002/advs.201900513 PMC6794619 31637157

[91] Pegtel DM, Gould SJ. Exosomes. Annu Rev Biochem. 2019;88:487–514. 31220978 10.1146/annurev-biochem-013118-111902

[92] Liu Y, Xue M, Han Y, Li Y, Xiao B, Wang W, Yu J, Ye X. Exosomes from M2c macrophages alleviate intervertebral disc degeneration by promoting synthesis of the extracellular matrix via MiR-124/CILP/TGF-β. Bioeng Transl Med. 2023;8(6):e10500. 38023721 10.1002/btm2.10500 PMC10658595

[93] Zhang S, Teo KYW, Chuah SJ, Lai RC, Lim SK, Toh WS. MSC exosomes alleviate temporomandibular joint osteoarthritis by attenuating inflammation and restoring matrix homeostasis. Biomaterials. 2019;200:35–47. 30771585 10.1016/j.biomaterials.2019.02.006

[94] He L, He T, Xing J, Zhou Q, Fan L, Liu C, Chen Y, Wu D, Tian Z, Liu B, et al. Bone marrow mesenchymal stem cell-derived exosomes protect cartilage damage and relieve knee osteoarthritis pain in a rat model of osteoarthritis. Stem Cell Res Ther. 2020;11(1):276. 32650828 10.1186/s13287-020-01781-w PMC7350730

[95] Hartman N, Loyal J, Fabi S. Update on exosomes in aesthetics. Dermatologic Surg. 2022;48(8):862–865. 10.1097/DSS.0000000000003487 35580250

[96] Wu J-Y, Wu S-N, Zhang L-P, Zhao X-S, Li Y, Yang Q-Y, Yuan R-Y, Liu J-L, Mao H-J, Zhu N-W. Stem cell-derived exosomes: A new method for reversing skin aging. Tissue Eng Regen Med. 2022;19(5):961–968. 35809187 10.1007/s13770-022-00461-5 PMC9477989

[97] Han M, Yang H, Lu X, Li Y, Liu Z, Li F, Shang Z, Wang X, Li X, Li J, et al. Three-dimensional-cultured MSC-derived exosome-hydrogel hybrid microneedle array patch for spinal cord repair. Nano Lett. 2022;22(15):6391–6401. 35876503 10.1021/acs.nanolett.2c02259

[98] Jiang D, Gong F, Ge X, Lv C, Huang C, Feng S, Zhou Z, Rong Y, Wang J, Ji C, et al. Neuron-derived exosomes-transmitted miR-124-3p protect traumatically injured spinal cord by suppressing the activation of neurotoxic microglia and astrocytes. J Nanobiotechnology. 2020;18(1):105. 32711535 10.1186/s12951-020-00665-8 PMC7382861

[99] Lin S, Chang Y, Lee W, Chiang C, Liu S, Lee H, Jeng L, Shyu W. Role of STAT3-FOXO3 signaling in the modulation of neuroplasticity by PD-L1-HGF-decorated mesenchymal stem cell-derived exosomes in a murine stroke model. Adv Sci. 2024;11(36):2404882. 10.1002/advs.202404882 PMC11423231 39049677

[100] Ji X, Zhou S, Wang N, Wang J, Wu Y, Duan Y, Ni P, Zhang J, Yu S. Cerebral-organoid-derived exosomes alleviate oxidative stress and promote LMX1A-dependent dopaminergic differentiation. Int J Mol Sci. 2023;24(13):11048. 37446226 10.3390/ijms241311048 PMC10341736

[101] Shen K, Jia Y, Wang X, Zhang J, Liu K, Wang J, Cai W, Li J, Li S, Zhao M, et al. Exosomes from adipose-derived stem cells alleviate the inflammation and oxidative stress via regulating Nrf2/HO-1 axis in macrophages. Free Radic Biol Med. 2021;165:54–66. 33476797 10.1016/j.freeradbiomed.2021.01.023

[102] Purushothaman A. Exosomes from cell culture-conditioned medium: Isolation by ultracentrifugation and characterization. Methods Mol Biol. 2019;1952:233–244. 30825179 10.1007/978-1-4939-9133-4_19

[103] Caradec J, Kharmate G, Hosseini-Beheshti E, Adomat H, Gleave M, Guns E. Reproducibility and efficiency of serum-derived exosome extraction methods. Clin Biochem. 2014;47(13-14):1286–1292. 24956264 10.1016/j.clinbiochem.2014.06.011

[104] Helwa I, Cai J, Drewry MD, Zimmerman A, Dinkins MB, Khaled ML, Seremwe M, Dismuke WM, Bieberich E, Stamer WD, et al. A comparative study of serum exosome isolation using differential ultracentrifugation and three commercial reagents. PLOS ONE. 2017;12(1): Article e0170628. 28114422 10.1371/journal.pone.0170628 PMC5256994

[105] D’Acunzo P, Kim Y, Ungania JM, Pérez-González R, Goulbourne CN, Levy E. Isolation of mitochondria-derived mitovesicles and subpopulations of microvesicles and exosomes from brain tissues. Nat Protoc. 2022;17(11):2517–2549. 35962195 10.1038/s41596-022-00719-1 PMC9633367

[106] Monguió Tortajada M, Gálvez Montón C, Bayes Genis A, Roura S, Borràs FE. Extracellular vesicle isolation methods: Rising impact of size-exclusion chromatography. Cell Mol Life Sci. 2019;76(12):2369–2382. 30891621 10.1007/s00018-019-03071-y PMC11105396

[107] Gao M, Cai J, Zitkovsky HS, Chen B, Guo L. Comparison of yield, purity, and functional properties of large-volume exosome isolation using ultrafiltration and polymer-based precipitation. Plast Reconstr Surg. 2022;149(3):638–649. 35196679 10.1097/PRS.0000000000008830

[108] Xiang X, Guan F, Jiao F, Li H, Zhang W, Zhang Y, Qin W. A new urinary exosome enrichment method by a combination of ultrafiltration and TiO(2) nanoparticles. Anal Methods. 2021;13(13):1591–1600. 33729255 10.1039/d1ay00102g

[109] Lin B, Lei Y, Wang J, Zhu L, Wu Y, Zhang H, Wu L, Zhang P, Yang C. Microfluidic-based exosome analysis for liquid biopsy. Small Methods. 2021;5(3):e2001131. 34927834 10.1002/smtd.202001131

[110] Wang J, Ma P, Kim DH, Liu B-F, Demirci U. Towards microfluidic-based exosome isolation and detection for tumor therapy. Nano Today. 2021;37:101066. 33777166 10.1016/j.nantod.2020.101066 PMC7990116

[111] Theel EK, Schwaminger SP. Microfluidic approaches for affinity-based exosome separation. Int J Mol Sci. 2022;23(16):9004. 36012270 10.3390/ijms23169004 PMC9409173

[112] Zhao W, Zhang L, Ye Y, Li Y, Luan X, Liu J, Cheng J, Zhao Y, Li M, Huang C. Microsphere mediated exosome isolation and ultra-sensitive detection on a dielectrophoresis integrated microfluidic device. Analyst. 2021;146(19):5962–5972. 34494041 10.1039/d1an01061a

[113] Antopolsky M, Chang M, Chang Y-J, Chao PY, Yu Q. Exosome purification based on PEG-coated Fe_3_O_4_ nanoparticles. PLOS ONE. 2018;13(6):e0199438. 29933408 10.1371/journal.pone.0199438 PMC6014651

[114] Sundaram PM, Casadei L, Lopez G, Braggio D, Balakirsky G, Pollock R, Prakash S. Multi-layer micro-nanofluidic device for isolation and capture of extracellular vesicles derived from liposarcoma cell conditioned media. J Microelectromech Syst. 2020;29(5):776–782. 33519169 10.1109/jmems.2020.3006786 PMC7839931

[115] Cai S, Luo B, Jiang P, Zhou X, Lan F, Yi Q, Wu Y. Immuno-modified superparamagnetic nanoparticles via host–guest interactions for high-purity capture and mild release of exosomes. Nanoscale. 2018;10(29):14280–14289. 30014056 10.1039/c8nr02871k

[116] Lim J, Choi M, Lee H, Kim Y-H, Han J-Y, Lee ES, Cho Y. Direct isolation and characterization of circulating exosomes from biological samples using magnetic nanowires. J. Nanobiotechnology. 2019;17(1):1. 30612562 10.1186/s12951-018-0433-3 PMC6322342

[117] Sidhom K, Obi PO, Saleem A. A review of exosomal isolation methods: Is size exclusion chromatography the best option? Int J Mol Sci. 2020;21(18):6466. 32899828 10.3390/ijms21186466 PMC7556044

[118] Kaddour H, Tranquille M, Okeoma CM. The past, the present, and the future of the size exclusion chromatography in extracellular vesicles separation. Viruses. 2021;13(11):2272. 34835078 10.3390/v13112272 PMC8618570

[119] Karimi N, Cvjetkovic A, Jang SC, Crescitelli R, Hosseinpour Feizi MA, Nieuwland R, Lötvall J, Lässer C. Detailed analysis of the plasma extracellular vesicle proteome after separation from lipoproteins. Cell Mol Life Sci. 2018;75(15):2873–2886. 29441425 10.1007/s00018-018-2773-4 PMC6021463

[120] Bai H, Wang X, Zhang B, Liu W. A comparison of size exclusion chromatography-based tandem strategies for plasma exosome enrichment and proteomic analysis. Anal Methods. 2023;15(45):6245–6251. 37955202 10.1039/d3ay01704d

[121] Miron RJ, Zhang Y. Understanding exosomes: Part 1—Characterization, quantification and isolation techniques. Periodontology. 2000;94(1):231–256. 10.1111/prd.12520 37740431

[122] Szatanek R, Baj-Krzyworzeka M, Zimoch J, Lekka M, Siedlar M, Baran J. The methods of choice for extracellular vesicles (EVs) characterization. Int J Mol Sci. 2017;18(6):1153. 28555055 10.3390/ijms18061153 PMC5485977

[123] Zhang W, Peng P, Kuang Y, Yang J, Cao D, You Y, Shen K. Characterization of exosomes derived from ovarian cancer cells and normal ovarian epithelial cells by nanoparticle tracking analysis. Tumour Biol. 2015;37(3):4213–4221. 26490993 10.1007/s13277-015-4105-8

[124] Corso G, Heusermann W, Trojer D, Görgens A, Steib E, Voshol J, Graff A, Genoud C, Lee Y, Hean J, et al. Systematic characterization of extracellular vesicle sorting domains and quantification at the single molecule—Single vesicle level by fluorescence correlation spectroscopy and single particle imaging. J Extracell Vesicles. 2019;8(1):1663034. 10.1080/20013078.2019.1663043 PMC6758720 31579435

[125] Sanaee M, Sandberg E, Ronquist KG, Morrell JM, Widengren J, Gallo K. Coincident fluorescence-burst analysis of the loading yields of exosome-mimetic nanovesicles with fluorescently-labeled cargo molecules. Small. 2022;18(12):e2106241. 35084110 10.1002/smll.202106241

[126] Huang Y, Liu Y, Huang Q, Sun S, Ji Z, Huang L, Li Z, Huang X, Deng W, Li T. TMT-based quantitative proteomics analysis of synovial fluid-derived exosomes in inflammatory arthritis. Front Immunol. 2022;13:800902. 35359923 10.3389/fimmu.2022.800902 PMC8961740

[127] Tao L, Zhou J, Yuan C, Zhang L, Li D, Si D, Xiu D, Zhong L. Metabolomics identifies serum and exosomes metabolite markers of pancreatic cancer. Metabolomics. 2019;15(6):86. 31147790 10.1007/s11306-019-1550-1

[128] Ma L, Yu H, Zhu Y, Xu K, Zhao A, Ding L, Gao H, Zhang M. Isolation and proteomic profiling of urinary exosomes from patients with colorectal cancer. Proteome Sci. 2023;21(1):3. 36759883 10.1186/s12953-023-00203-y PMC9909931

[129] van der Pol E, Coumans FAW, Grootemaat AE, Gardiner C, Sargent IL, Harrison P, Sturk A, van Leeuwen TG, Nieuwland R. Particle size distribution of exosomes and microvesicles determined by transmission electron microscopy, flow cytometry, nanoparticle tracking analysis, and resistive pulse sensing. J Thromb Haemost. 2014;12(7):1182–1192. 24818656 10.1111/jth.12602

[130] Ramos JW, Lyu TS, Ahn Y, Im Y-J, Kim S-S, Lee K-H, Kim J, Choi Y, Lee D, Kang E, et al. The characterization of exosomes from fibrosarcoma cell and the useful usage of dynamic light scattering (DLS) for their evaluation. PLOS ONE. 2021;16(1):e0231994. 33497388 10.1371/journal.pone.0231994 PMC7837462

[131] Ono K, Okusha Y, Tran MT, Umemori K, Eguchi T. Western blot protocols for analysis of CCN proteins and fragments in exosomes, vesicle-free fractions, and cells. Methods Mol Biol. 2023;2582:39–57. 36370343 10.1007/978-1-0716-2744-0_5

[132] Logozzi M, Di Raimo R, Mizzoni D, Fais S. Immunocapture-based ELISA to characterize and quantify exosomes in both cell culture supernatants and body fluids. Methods Enzymol. 2020;645:155–180. 33565970 10.1016/bs.mie.2020.06.011 PMC7346819

[133] Iha K, Tsurusawa N, Tsai H-Y, Lin M-W, Sonoda H, Watabe S, Yoshimura T, Ito E. Ultrasensitive ELISA detection of proteins in separated lumen and membrane fractions of cancer cell exosomes. Anal Biochem. 2022;654:114831. 35921878 10.1016/j.ab.2022.114831

[134] Malenica M, Vukomanović M, Kurtjak M, Masciotti V, Dal Zilio S, Greco S, Lazzarino M, Krušić V, Perčić M, Jelovica Badovinac I. Perspectives of microscopy methods for morphology characterisation of extracellular vesicles from human biofluids. Biomedicines. 2021;9(6):603. 34073297 10.3390/biomedicines9060603 PMC8228884

[135] Yang B, Chen Y, Shi J. Exosome biochemistry and advanced nanotechnology for next-generation theranostic platforms. Adv Mater. 2019;31(2):1802896. 10.1002/adma.201802896 30126052

[136] Lei L, Pan W, Shou X, Shao Y, Ye S, Zhang J, Kolliputi N, Shi L. Nanomaterials-assisted gene editing and synthetic biology for optimizing the treatment of pulmonary diseases. J Nanobiotechnology. 2024;22(1):343. 38890749 10.1186/s12951-024-02627-w PMC11186260

[137] Chen Z, Xiong M, Tian J, Song D, Duan S, Zhang L. Encapsulation and assessment of therapeutic cargo in engineered exosomes: A systematic review. J Nanobiotechnology. 2024;22(1):18. 38172932 10.1186/s12951-023-02259-6 PMC10765779

[138] Liang YJ, Xu X, Xu LM, Iqbal Z, Ouyang K, Zhang HW, Wen CY, Duan L, Xia J. Chondrocyte-specific genomic editing enabled by hybrid exosomes for osteoarthritis treatment. Theranostics. 2022;12(11):4866–4878. 35836795 10.7150/thno.69368 PMC9274754

[139] Du J, Wan Z, Wang C, Lu F, Wei M, Wang D, Hao Q. Designer exosomes for targeted and efficient ferroptosis induction in cancer via chemo-photodynamic therapy. Theranostics. 2021;11(17):8185–8196. 34373736 10.7150/thno.59121 PMC8344009

[140] Kang JY, Mun D, Chun Y, Park D, Kim H, Yun NR, Joung B. Engineered small extracellular vesicle-mediated NOX4 siRNA delivery for targeted therapy of cardiac hypertrophy. J Extracellular Vesicles. 2023;12(10):e12371. 37795828 10.1002/jev2.12371 PMC10552075

[141] Yang Z, Shi J, Xie J, Wang Y, Sun J, Liu T, Zhao Y, Zhao X, Wang X, Ma Y, et al. Large-scale generation of functional mRNA-encapsulating exosomes via cellular nanoporation. Nat Biomed Eng. 2020;4(1):69–83. 31844155 10.1038/s41551-019-0485-1 PMC7080209

[142] Shi Y, Guo S, Liang Y, Liu L, Wang A, Sun K, Li Y. Construction and evaluation of liraglutide delivery system based on milk exosomes: A new idea for oral peptide delivery. Curr Pharm Biotechnol. 2022;23(8):1072–1079. 34414872 10.2174/1389201022666210820114236

[143] Evers MJW, van de Wakker SI, de Groot EM, de Jong OG, Gitz-François JJJ, Seinen CS, Sluijter JPG, Schiffelers RM, Vader P. Functional siRNA delivery by extracellular vesicle–liposome hybrid nanoparticles. Adv Healthc Mater. 2021;11(5):e2101202. 34382360 10.1002/adhm.202101202 PMC11468224

[144] Sato YT, Umezaki K, Sawada S, Mukai S-A, Sasaki Y, Harada N, Shiku H, Akiyoshi K. Engineering hybrid exosomes by membrane fusion with liposomes. Sci Rep. 2016;6(1):231933. 10.1038/srep21933 PMC4766490 26911358

[145] Li L, He D, Guo Q, Zhang Z, Ru D, Wang L, Gong K, Liu F, Duan Y, Li H. Exosome-liposome hybrid nanoparticle codelivery of TP and miR497 conspicuously overcomes chemoresistant ovarian cancer. J Nanobiotechnology. 2022;20(1):50. 35078498 10.1186/s12951-022-01264-5 PMC8787930

[146] Estes S, Konstantinov K, Young JD. Manufactured extracellular vesicles as human therapeutics: Challenges, advances, and opportunities. Curr Opin Biotechnol. 2022;77: Article 102776. 36041354 10.1016/j.copbio.2022.102776

[147] Hu W, Wang W, Chen Z, Chen Y, Wang Z. Engineered exosomes and composite biomaterials for tissue regeneration. Theranostics. 2024;14(5):2099–2126. 38505616 10.7150/thno.93088 PMC10945329

[148] Mishra S, Ganguli M. Functions of, and replenishment strategies for, chondroitin sulfate in the human body. Drug Discov Today. 2021;26(5):1185–1199. 33549530 10.1016/j.drudis.2021.01.029

[149] Nikhil A, Kumar A. Evaluating potential of tissue-engineered cryogels and chondrocyte derived exosomes in articular cartilage repair. Biotechnol Bioeng. 2022;119(2):605–625. 34723385 10.1002/bit.27982

[150] Bright LME, Griffin L, Mondal A, Hopkins S, Ozkan E, Handa H. Biomimetic gasotransmitter-releasing alginate beads for biocompatible antimicrobial therapy. J Colloid Interface Sci. 2022;628:911–921. 36030716 10.1016/j.jcis.2022.08.113 PMC9728620

[151] Liang Y, Shuai Q, Zhang X, Jin S, Guo Y, Yu Z, Xu X, Ao R, Peng Z, Lv H, et al. Incorporation of decidual stromal cells derived exosomes in sodium alginate hydrogel as an innovative therapeutic strategy for advancing endometrial regeneration and reinstating fertility. Adv Healthc Mater. 2024;(13):13, 2303674. 10.1002/adhm.202303674 38315148

[152] Zhang YM, Cai ZC, Shen YL, Lu QZ, Gao W, Zhong X, Yao K, Yuan J, Liu HB. Hydrogel-load exosomes derived from dendritic cells improve cardiac function via Treg cells and the polarization of macrophages following myocardial infarction. J Nanobiotechnology. 2021;19(1):271. 34496871 10.1186/s12951-021-01016-x PMC8424987

[153] Gan JJ, Sun LY, Chen GP, Ma WJ, Zhao YJ, Sun LY. Mesenchymal stem cell exosomes encapsulated oral microcapsules for acute colitis treatment. Adv Healthc Mater. 2022;11(17):e2201105. 35737997 10.1002/adhm.202201105

[154] Sun M, Li Q, Yu H, Cheng J, Wu N, Shi W, Zhao F, Shao Z, Meng Q, Chen H, et al. Cryo-self-assembled silk fibroin sponge as a biodegradable platform for enzyme-responsive delivery of exosomes. Bioact Mater. 2022;8:505–514. 34541416 10.1016/j.bioactmat.2021.06.017 PMC8433120

[155] Zhou Z, Cui J, Wu S, Geng Z, Su J. Silk fibroin-based biomaterials for cartilage/osteochondral repair. Theranostics. 2022;12(11):5103. 35836802 10.7150/thno.74548 PMC9274741

[156] Wani SUD, Gautam SP, Qadrie ZL, Gangadharappa HV. Silk fibroin as a natural polymeric based bio-material for tissue engineering and drug delivery systems—A review. Int J Biol Macromol. 2020;163:2145–2161. 32950527 10.1016/j.ijbiomac.2020.09.057

[157] Rui K, Tang X, Shen Z, Jiang C, Zhu Q, Liu S, Che N, Tian J, Ling J, Yang Y. Exosome inspired photo-triggered gelation hydrogel composite on modulating immune pathogenesis for treating rheumatoid arthritis. J Nanobiotechnology. 2023;21(1):111. 36973764 10.1186/s12951-023-01865-8 PMC10044428

[158] Han C, Zhou J, Liu B, Liang C, Pan X, Zhang Y, Zhang Y, Wang Y, Shao L, Zhu B, et al. Delivery of miR-675 by stem cell-derived exosomes encapsulated in silk fibroin hydrogel prevents aging-induced vascular dysfunction in mouse hindlimb. Mater Sci Eng C Mater Biol Appl. 2019;99:322–332. 30889706 10.1016/j.msec.2019.01.122

[159] Li Q, Hu W, Huang Q, Yang J, Li B, Ma K, Wei Q, Wang Y, Su J, Sun M, et al. MiR146a-loaded engineered exosomes released from silk fibroin patch promote diabetic wound healing by targeting IRAK1. Signal Transduct Target Ther. 2023;8(1):62. 36775818 10.1038/s41392-022-01263-w PMC9922687

[160] Deng H, Wang J, An R. Hyaluronic acid-based hydrogels: As an exosome delivery system in bone regeneration. Front Pharmacol. 2023;14:1131001. 37007032 10.3389/fphar.2023.1131001 PMC10063825

[161] Vasvani S, Kulkarni P, Rawtani D. Hyaluronic acid: A review on its biology, aspects of drug delivery, route of administrations and a special emphasis on its approved marketed products and recent clinical studies. Int J Biol Macromol. 2020;151:1012–1029. 31715233 10.1016/j.ijbiomac.2019.11.066

[162] Liu K, Chen C, Zhang H, Chen Y, Zhou S. Adipose stem cell-derived exosomes in combination with hyaluronic acid accelerate wound healing through enhancing re-epithelialization and vascularization. Br J Dermatol. 2019;181(4):854–856. 30953591 10.1111/bjd.17984

[163] Derkus B. Human cardiomyocyte-derived exosomes induce cardiac gene expressions in mesenchymal stromal cells within 3D hyaluronic acid hydrogels and in dose-dependent manner. J Mater Sci Mater Med. 2021;32(1):2. 33469781 10.1007/s10856-020-06474-7 PMC7815535

[164] He J, Hu X, Cao J, Zhang Y, Xiao J, Peng J, Chen D, Xiong C, Zhang L. Chitosan-coated hydroxyapatite and drug-loaded polytrimethylene carbonate/polylactic acid scaffold for enhancing bone regeneration. Carbohydr Polym. 2021;253:117198. 33278972 10.1016/j.carbpol.2020.117198

[165] Gholap AD, Rojekar S, Kapare HS, Vishwakarma N, Raikwar S, Garkal A, Mehta TA, Jadhav H, Prajapati MK, Annapure U. Chitosan scaffolds: Expanding horizons in biomedical applications. Carbohydr Polym. 2024;323:121394. 37940287 10.1016/j.carbpol.2023.121394

[166] Bahar D, Gonen ZB, Gumusderelioglu M, Onger ME, Tokak EK, Ozturk-Kup F, Ozkan BB, Gokdemir NS, Cetin M. Repair of rat calvarial bone defect by using exosomes of umbilical cord-derived mesenchymal stromal cells embedded in chitosan/hydroxyapatite scaffolds. Int J Oral Maxillofac Implants. 2022;37(5):943–950. 36170309 10.11607/jomi.9515

[167] Liu XY, Feng YH, Feng QB, Zhang JY, Zhong L, Liu P, Wang S, Huang YR, Chen XY, Zhou LX. Low-temperature 3D-printed collagen/chitosan scaffolds loaded with exosomes derived from neural stem cells pretreated with insulin growth factor-1 enhance neural regeneration after traumatic brain injury. Neural Regen Res. 2023;18(9):1990–1998. 36926724 10.4103/1673-5374.366497 PMC10233754

[168] Abu Hajleh MN, Al-Samydai A, Al-Dujaili EAS. Nano, micro particulate and cosmetic delivery systems of polylactic acid: A mini review. J Cosmet Dermatol. 2020;19(11):2805–2811. 32954588 10.1111/jocd.13696

[169] Gandolfi MG, Gardin C, Zamparini F, Ferroni L, Esposti MD, Parchi G, Ercan B, Manzoli L, Fava F, Fabbri P, et al. Mineral-doped poly(L-lactide) acid scaffolds enriched with exosomes improve osteogenic commitment of human adipose-derived mesenchymal stem cells. Nano. 2020;10(3):432. 10.3390/nano10030432 PMC7153699 32121340

[170] Zhang Y, Huo M, Wang Y, Xiao L, Wu J, Ma Y, Zhang D, Lang X, Wang X. A tailored bioactive 3D porous poly(lactic-acid)-exosome scaffold with osteo-immunomodulatory and osteogenic differentiation properties. J Biol Eng. 2022;16(1):22. 35996115 10.1186/s13036-022-00301-z PMC9394013

[171] Han L, Liu H, Fu H, Hu Y, Fang W, Liu J. Exosome-delivered BMP-2 and polyaspartic acid promotes tendon bone healing in rotator cuff tear via Smad/RUNX2 signaling pathway. Bioengineered. 2022;13(1):1459–1475. 35258414 10.1080/21655979.2021.2019871 PMC8805918

[172] Wang H, Zhao Z, Liu Y, Shao C, Bian F, Zhao Y. Biomimetic enzyme cascade reaction system in microfluidic electrospray microcapsules. Sci Adv. 2018;4(6):eaat2816. 29922720 10.1126/sciadv.aat2816 PMC6003728

[173] Lin J, Wang Z, Huang J, Tang S, Saiding Q, Zhu Q, Cui W. Microenvironment-protected exosome-hydrogel for facilitating endometrial regeneration, fertility restoration, and live birth of offspring. Small. 2021;17(11):e2007235. 33590681 10.1002/smll.202007235

[174] Yang L, Li W, Zhao Y, Shang L. Magnetic polysaccharide mesenchymal stem cells exosomes delivery microcarriers for synergistic therapy of osteoarthritis. ACS Nano. 2024; 10.1021/acsnano.4c01406. 10.1021/acsnano.4c01406 39039744

[175] Zhang Y, Li M, Wang Y, Han F, Shen K, Luo L, Li Y, Jia Y, Zhang J, Cai W, et al. Exosome/metformin-loaded self-healing conductive hydrogel rescues microvascular dysfunction and promotes chronic diabetic wound healing by inhibiting mitochondrial fission. Bioact Mater. 2023;26:323–336. 36950152 10.1016/j.bioactmat.2023.01.020 PMC10027478

[176] Yang J, Chen Z, Pan D, Li H, Shen J. Umbilical cord-derived mesenchymal stem cell-derived exosomes combined pluronic F127 hydrogel promote chronic diabetic wound healing and complete skin regeneration. Int J Nanomedicine. 2020;15:5911–5926. 32848396 10.2147/IJN.S249129 PMC7429232

[177] Guan P, Liu C, Xie D, Mao S, Ji Y, Lin Y, Chen Z, Wang Q, Fan L, Sun Y. Exosome-loaded extracellular matrix-mimic hydrogel with anti-inflammatory property facilitates/promotes growth plate injury repair. Bioact Mater. 2022;10:145–158. 34901536 10.1016/j.bioactmat.2021.09.010 PMC8637006

[178] Khayambashi P, Iyer J, Pillai S, Upadhyay A, Zhang Y, Tran S. Hydrogel encapsulation of mesenchymal stem cells and their derived exosomes for tissue engineering. Int J Mol Sci. 2021;22(2):684. 33445616 10.3390/ijms22020684 PMC7827932

[179] Wang C, Wang M, Xu T, Zhang X, Lin C, Gao W, Xu H, Lei B, Mao C. Engineering bioactive self-healing antibacterial exosomes hydrogel for promoting chronic diabetic wound healing and complete skin regeneration. Theranostics. 2019;9(1):65–76. 30662554 10.7150/thno.29766 PMC6332800

[180] Gu X, Liu Y, Chen G, Wang H, Shao C, Chen Z, Lu P, Zhao Y. Mesoporous colloidal photonic crystal particles for intelligent drug delivery. ACS Appl Mater Interfaces. 2018;10(40):33936–33944. 30215247 10.1021/acsami.8b11175

[181] Yang L, Li W, Zhao Y, Wang Y, Shang L. Stem cell recruitment polypeptide hydrogel microcarriers with exosome delivery for osteoarthritis treatment. J Nanobiotechnology. 2024;22(1):512. 39192268 10.1186/s12951-024-02765-1 PMC11348651

[182] Gao Y, Yuan Z, Yuan X, Wan Z, Yu Y, Zhan Q, Zhao Y, Han J, Huang J, Xiong C, et al. Bioinspired porous microspheres for sustained hypoxic exosomes release and vascularized bone regeneration. Bioact Mater. 2022;14:377–388. 35386817 10.1016/j.bioactmat.2022.01.041 PMC8964815

[183] Li H, Wang X, Guo X, Wan Q, Teng Y, Liu J. Development of rapamycin-encapsulated exosome-mimetic nanoparticles-in-PLGA microspheres for treatment of hemangiomas. Biomed Pharmacother. 2022;148: Article 112737. 35276517 10.1016/j.biopha.2022.112737

[184] Yang Y, Zheng W, Tan W, Wu X, Dai Z, Li Z, Yan Z, Ji Y, Wang Y, Su W, et al. Injectable MMP1-sensitive microspheres with spatiotemporally controlled exosome release promote neovascularized bone healing. Acta Biomater. 2023;157:321–336. 36481504 10.1016/j.actbio.2022.11.065

[185] Chen P, Ning X, Li W, Pan Y, Wang L, Li H, Fan X, Zhang J, Luo T, Wu Y, et al. Fabrication of Tβ4-exosome-releasing artificial stem cells for myocardial infarction therapy by improving coronary collateralization. Bioact Mater. 2022;14:416–429. 35386821 10.1016/j.bioactmat.2022.01.029 PMC8964820

[186] Fathi-Karkan S, Heidarzadeh M, Narmi MT, Mardi N, Amini H, Saghati S, Abrbekoh FN, Saghebasl S, Rahbarghazi R, Khoshfetrat AB. Exosome-loaded microneedle patches: Promising factor delivery route. Int J Biol Macromol. 2023;243:125232. 37302628 10.1016/j.ijbiomac.2023.125232

[187] Song K, Hao Y, Tan X, Huang H, Wang L, Zheng W. Microneedle-mediated delivery of ziconotide-loaded liposomes fused with exosomes for analgesia. J Control Release. 2023;356:448–462. 36898532 10.1016/j.jconrel.2023.03.007

[188] Yang G, Chen Q, Wen D, Chen Z, Wang J, Chen G, Wang Z, Zhang X, Zhang Y, Hu Q, et al. A therapeutic microneedle patch made from hair-derived keratin for promoting hair regrowth. ACS Nano. 2019;13(4):4354–4360. 30942567 10.1021/acsnano.8b09573

[189] Zeng J, Sun Z, Zeng F, Gu C, Chen X. M2 macrophage-derived exosome-encapsulated microneedles with mild photothermal therapy for accelerated diabetic wound healing. Mater Today Bio. 2023;20: Article 100649. 10.1016/j.mtbio.2023.100649 PMC10189292 37206877

[190] Liu A, Wang Q, Zhao Z, Wu R, Wang M, Li J, Sun K, Sun Z, Lv Z, Xu J, et al. Nitric oxide nanomotor driving exosomes-loaded microneedles for Achilles tendinopathy healing. ACS Nano. 2021;15(8):13339–13350. 34324304 10.1021/acsnano.1c03177

[191] Shi Y, Zhao J, Li H, Yu M, Zhang W, Qin D, Qiu K, Chen X, Kong M, Drug-Free A. Hair follicle cycling regulatable, separable, antibacterial microneedle patch for hair regeneration therapy. Adv Healthc Mater. 2022;11(19):e2200908. 35817085 10.1002/adhm.202200908

[192] Wang B, Qinglai T, Yang Q, Li M, Zeng S, Yang X, Xiao Z, Tong X, Lei L, Li S. Functional acellular matrix for tissue repair. Mater Today Bio. 2023;18: Article 100530. 10.1016/j.mtbio.2022.100530 PMC9806685 36601535

[193] Yang P, Ju Y, Hu Y, Xie X, Fang B, Lei L. Emerging 3D bioprinting applications in plastic surgery. Biomater Res. 2023;27(1):1. 36597149 10.1186/s40824-022-00338-7 PMC9808966

[194] Li Q, Yu H, Zhao F, Cao C, Wu T, Fan Y, Ao Y, Hu X. 3D printing of microenvironment-specific bioinspired and exosome-reinforced hydrogel scaffolds for efficient cartilage and subchondral bone regeneration. Adv Sci. 2023;10(26): Article e2303650. 10.1002/advs.202303650 PMC10502685 37424038

[195] Chen P, Zheng L, Wang Y, Tao M, Xie Z, Xia C, Gu C, Chen J, Qiu P, Mei S, et al. Desktop-stereolithography 3D printing of a radially oriented extracellular matrix/mesenchymal stem cell exosome bioink for osteochondral defect regeneration. Theranostics. 2019;9(9):2439–2459. 31131046 10.7150/thno.31017 PMC6525998

[196] Kang Y, Xu C, Meng L, Dong XF, Qi M, Jiang DQ. Exosome-functionalized magnesium-organic framework-based scaffolds with osteogenic, angiogenic and anti-inflammatory properties for accelerated bone regeneration. Bioact Mater. 2022;18:26–41. 35387167 10.1016/j.bioactmat.2022.02.012 PMC8961306

[197] Su Y, Gao Q, Deng R, Zeng L, Guo J, Ye B, Yu J, Guo X. Aptamer engineering exosomes loaded on biomimetic periosteum to promote angiogenesis and bone regeneration by targeting injured nerves via JNK3 MAPK pathway. Mater Today Bio. 2022;16: Article 100434. 10.1016/j.mtbio.2022.100434 PMC9519612 36186848

[198] Aringer M. Inflammatory markers in systemic lupus erythematosus. J Autoimmun. 2020;110:102374. 31812331 10.1016/j.jaut.2019.102374

[199] Morand EF, Fernandez-Ruiz R, Blazer A, Niewold TB. Advances in the management of systemic lupus erythematosus. BMJ. 2023;383:e073980. 37884289 10.1136/bmj-2022-073980

[200] Chuang H, Chen M, Chen Y, Yang H, Ciou Y, Hsueh C, Tsai C, Tan T. BPI overexpression suppresses Treg differentiation and induces exosome-mediated inflammation in systemic lupus erythematosus. Theranostics. 2021;11(20):9953–9966. 34815797 10.7150/thno.63743 PMC8581436

[201] Zhang M, Johnson-Stephenson TK, Wang W, Wang Y, Li J, Li L, Zen K, Chen X, Zhu D. Mesenchymal stem cell-derived exosome-educated macrophages alleviate systemic lupus erythematosus by promoting efferocytosis and recruitment of IL-17^+^ regulatory T cell. Stem Cell Res Ther. 2022;13(1):484. 36153633 10.1186/s13287-022-03174-7 PMC9509559

[202] Dou R, Zhang X, Xu X, Wang P, Yan B. Mesenchymal stem cell exosomal tsRNA-21109 alleviate systemic lupus erythematosus by inhibiting macrophage M1 polarization. Mol Immunol. 2021;139:106–114. 34464838 10.1016/j.molimm.2021.08.015

[203] Wang W, Yue C, Gao S, Li S, Zhou J, Chen J, Fu J, Sun W, Hua C. Promising roles of exosomal microRNAs in systemic lupus erythematosus. Front Immunol. 2021;12: Article 757096. 34966383 10.3389/fimmu.2021.757096 PMC8710456

[204] Zhao Y, Song W, Yuan Z, Li M, Wang G, Wang L, Liu Y, Diao B. Exosome derived from human umbilical cord mesenchymal cell exerts immunomodulatory effects on B cells from SLE patients. J Immunol Res. 2023;12:3177584. 10.1155/2023/3177584 PMC10198761 37215068

[205] Torres T, Ferreira EO, Gonçalo M, Mendes-Bastos P, Selores M, Filipe P. Update on atopic dermatitis. Acta Medica Port. 2019;32(9):606–613. 10.20344/amp.11963 31493365

[206] Ungar B, Hartzell S, Lozano-Ojalvo D, Ghalili S, Bose S, Golant AK, Tan K, Estrada YD, Singer GK, Pavel AB, et al. The impact of dupilumab treatment on SARS-CoV-2 T cell responses in atopic dermatitis patients. Allergy. 2023;78(2):571–574. 36181718 10.1111/all.15540 PMC9537998

[207] Najera J, Hao J. Recent advance in mesenchymal stem cells therapy for atopic dermatitis. J Cell Biochem. 2022;124(2):181–187. 36576973 10.1002/jcb.30365

[208] Shi C, Pei S, Ding Y, Tao C, Zhu Y, Peng Y, Li W, Yi Y. Exosomes with overexpressed miR 147a suppress angiogenesis and inflammatory injury in an experimental model of atopic dermatitis. Sci Rep. 2023;13(1):8904. 37264030 10.1038/s41598-023-34418-y PMC10235063

[209] Cho BS, Kim JO, Ha DH, Yi YW. Exosomes derived from human adipose tissue-derived mesenchymal stem cells alleviate atopic dermatitis. Stem Cell Res Ther. 2018;9(1):187. 29996938 10.1186/s13287-018-0939-5 PMC6042362

[210] Yoon J, Lee SK, Park A, Lee J, Jung I, Song KB, Choi EJ, Kim S, Yu J. Exosome from IFN-γ-primed induced pluripotent stem cell-derived mesenchymal stem cells improved skin inflammation and barrier function. Int J Mol Sci. 2023;24(14):11635. 37511392 10.3390/ijms241411635 PMC10380988

[211] Griffiths CEM, Armstrong AW, Gudjonsson JE, Barker JNWN. Psoriasis. Lancet. 2021;397(10281):1301–1315. 33812489 10.1016/S0140-6736(20)32549-6

[212] Zhang B, Lai RC, Sim WK, Choo ABH, Lane EB, Lim SK. Topical application of mesenchymal stem cell exosomes alleviates the imiquimod induced psoriasis-like inflammation. Int J Mol Sci. 2021;22(2):720. 33450859 10.3390/ijms22020720 PMC7828312

[213] Shao S, Fang H, Zhang J, Jiang M, Xue K, Ma J, Zhang J, Lei J, Zhang Y, Li B, et al. Neutrophil exosomes enhance the skin autoinflammation in generalized pustular psoriasis via activating keratinocytes. FASEB J. 2019;33(6):6813–6828. 30811955 10.1096/fj.201802090RR

[214] Jia H, Liu T, Yang Q, Zheng H, Fu S, Hong J, Zhou Z, Huang Q, Zhang Z, Zhang H, et al. Tumor-derived PD-L1+ exosomes with natural inflammation tropism for psoriasis-targeted treatment. Bioconjug Chem. 2023;34(4):809–824. 10.1021/acs.bioconjchem.3c00129 37036892

[215] Liu S, Gong J, Li T. miR-124-3p delivered using exosomes attenuates the keratinocyte response to IL-17A stimulation in psoriasis. Oxidative Med Cell Longev. 2022;2022:6264474. 10.1155/2022/6264474 PMC9581689 36275890

[216] Strnadova K, Sandera V, Dvorankova B, Kodet O, Duskova M, Smetana K, Lacina L. Skin aging: The dermal perspective. Clin Dermatol. 2019;37(4):326–335. 31345320 10.1016/j.clindermatol.2019.04.005

[217] Csekes E, Račková L. Skin aging, cellular senescence and natural polyphenols. Int J Mol Sci. 2021;22(23):12641. 34884444 10.3390/ijms222312641 PMC8657738

[218] Lv J, Yang S, Lv M, Lv J, Sui Y, Guo S. Protective roles of mesenchymal stem cells on skin photoaging: A narrative review. Tissue Cell. 2022;76:101746. 35182986 10.1016/j.tice.2022.101746

[219] Jin S, Wang Y, Wu X, Li Z, Zhu L, Niu Y, Zhou Y, Liu Y. Young exosome bio-nanoparticles restore aging-impaired tendon stem/progenitor cell function and reparative capacity. Adv Mater. 2023;35(18):2211602. 10.1002/adma.202211602 36779444

[220] Hu S, Li Z, Cores J, Huang K, Su T, Dinh P-U, Cheng K. Needle-free injection of exosomes derived from human dermal fibroblast spheroids ameliorates skin photoaging. ACS Nano. 2019;13(10):11273–11282. 31449388 10.1021/acsnano.9b04384 PMC7032013

[221] Xia W, Li M, Jiang X, Huang X, Gu S, Ye J, Zhu L, Hou M, Zan T. Young fibroblast-derived exosomal microRNA-125b transfers beneficial effects on aged cutaneous wound healing. J Nanobiotechnology. 2022;20(1):144. 35305652 10.1186/s12951-022-01348-2 PMC9744129

[222] Chen S, He Z, Xu J. Application of adipose-derived stem cells in photoaging: Basic science and literature review. Stem Cell Res Ther. 2020;11(1):491. 33225962 10.1186/s13287-020-01994-z PMC7682102

[223] Guo J-A, Yu P-J, Yang D-Q, Chen W, Maioli M. The antisenescence effect of exosomes from human adipose-derived stem cells on skin fibroblasts. Biomed Res Int. 2022;2022:1034316. 35813225 10.1155/2022/1034316 PMC9259368

[224] Yasin ZAM, Ibrahim F, Rashid NN, Razif MFM, Yusof R. The importance of some plant extracts as skin anti-aging resources: A review. Curr Pharm Biotechnol. 2018;18(11):864–876. 10.2174/1389201019666171219105920 29256348

[225] Trentini M, Zanolla I, Zanotti F, Tiengo E, Licastro D, Dal Monego S, Lovatti L, Zavan B. Apple derived exosomes improve collagen type I production and decrease MMPs during aging of the skin through downregulation of the NF-κB pathway as mode of action. Cells. 2022;11(24):3950. 36552714 10.3390/cells11243950 PMC9776931

[226] Lu L, Bai W, Wang M, Han C, Du H, Wang N, Gao M, Li D, Dong F, Ge X. Novel roles of bovine milk-derived exosomes in skin antiaging. J Cosmet Dermatol. 2024;23(4):1374–1385. 38105431 10.1111/jocd.16112

[227] Han G, Kim H, Kim DE, Ahn Y, Kim J, Jang YJ, Kim K, Yang Y, Kim SH. The potential of bovine colostrum-derived exosomes to repair aged and damaged skin cells. Pharmaceutics. 2022;14(2):307. 35214040 10.3390/pharmaceutics14020307 PMC8877896

[228] Sorg H, Sorg CGG. Skin wound healing: Of players, patterns, and processes. Eur Surg Res. 2023;64(2):141–157. 36417847 10.1159/000528271

[229] Zhong Z, Li Y, Sun Z, Wu X, Li J, Jiang S, Wang Y, Wang J, Du Y, Zhang S. Hypoxia-triggered exosome-mimetics accelerate vascularized osteogenesis. Mater Today. 2024;73:16–29.

[230] Liao Y, Zhang Z, Ouyang L, Mi B, Liu G. Engineered extracellular vesicles in wound healing: Design, paradigms, and clinical application. Small. 2023;20(7):2307058. 10.1002/smll.202307058 37806763

[231] Yang P, Ju Y, Liu X, Li Z, Liu H, Yang M, Chen X, Lei L, Fang B. Natural self-healing injectable hydrogels loaded with exosomes and berberine for infected wound healing. Mater Today Bio. 2023;23: Article 100875. 10.1016/j.mtbio.2023.100875 PMC10701414 38075251

[232] An Y, Lin S, Tan X, Zhu S, Nie F, Zhen Y, Gu L, Zhang C, Wang B, Wei W, et al. Exosomes from adipose-derived stem cells and application to skin wound healing. Cell Prolif. 2021;54(3):e12993. 33458899 10.1111/cpr.12993 PMC7941238

[233] Lee JH, Won YJ, Kim H, Choi M, Lee E, Ryoou B, Lee S-G, Cho BS. Adipose tissue-derived mesenchymal stem cell-derived exosomes promote wound healing and tissue regeneration. Int J Mol Sci. 2023;24(13):e2304023. 10.3390/ijms241310434 PMC10341478 37445612

[234] Kwak G, Cheng J, Kim H, Song S, Lee SJ, Yang Y, Jeong JH, Lee JE, Messersmith PB, Kim SH. Sustained exosome-guided macrophage polarization using hydrolytically degradable peg hydrogels for cutaneous wound healing: Identification of key proteins and miRNAs, and sustained release formulation. Small. 2022;18(15):e2200060. 35229462 10.1002/smll.202200060

[235] Xiong Y, Chen L, Liu P, Yu T, Lin C, Yan C, Hu Y, Zhou W, Sun Y, Panayi AC, et al. All-in-one: Multifunctional hydrogel accelerates oxidative diabetic wound healing through timed-release of exosome and fibroblast growth factor. Small. 2022;18(1):e2104229. 34791802 10.1002/smll.202104229

[236] Worrell JC, O'Reilly S. Bi-directional communication: Conversations between fibroblasts and immune cells in systemic sclerosis. J Autoimmun. 2020;113: Article 102526. 32713676 10.1016/j.jaut.2020.102526

[237] Liu Y-X, Sun J-M, Ho C-K, Gao Y, Wen D-S, Liu Y-D, Huang L, Zhang Y-F. Advancements in adipose-derived stem cell therapy for skin fibrosis. World J Stem Cells. 2023;15(5):342–353. 37342214 10.4252/wjsc.v15.i5.342 PMC10277960

[238] Zhuang XF, Hu X, Zhang SR, Li XM, Yuan XY, Wu YH. Mesenchymal stem cell-based therapy as a new approach for the treatment of systemic sclerosis. Clin Rev Allergy Immunol. 2023;64(3):284–320. 35031958 10.1007/s12016-021-08892-z

[239] Yu Y, Shen L, Xie X, Zhao J, Jiang M. The therapeutic effects of exosomes derived from human umbilical cord mesenchymal stem cells on scleroderma. Tissue Eng Regen Med. 2022;19(1):141–150. 34784013 10.1007/s13770-021-00405-5 PMC8782977

[240] Wang L, Li T, Ma X, Li Y, Li Z, Li Z, Yu N, Huang J, Han Q, Long X. Exosomes from human adipose–derived mesenchymal stem cells attenuate localized scleroderma fibrosis by the let-7a-5p/TGF-βR1/Smad axis. J Dermatol Sci. 2023;112(1):31–38. 37743142 10.1016/j.jdermsci.2023.09.001

[241] Xie L, Long X, Mo M, Jiang J, Zhang Q, Long M, Li M. Bone marrow mesenchymal stem cell-derived exosomes alleviate skin fibrosis in systemic sclerosis by inhibiting the IL-33/ST2 axis via the delivery of microRNA-214. Mol Immunol. 2023;157:146–157. 37028129 10.1016/j.molimm.2023.03.017

